# Machine learning optimized design of THz piezoelectric perovskite-based biosensor for the detection of formalin in aqueous environments

**DOI:** 10.1038/s41598-025-88766-y

**Published:** 2025-02-06

**Authors:** Jacob Wekalao, Shobhit K. Patel, Om Prakash Kumar, Fahad Ahmed Al-zahrani

**Affiliations:** 1 Department of Optics and Optical Engineering, University of Science and Technology of China, Hefei, 230026 China; 2 Department of Computer Engineering, Marwadi University, Rajkot, 360003 India; 3 Department of Electronics and Communication Engineering, Manipal Institute of Technology, Manipal Academy of Higher Education, Manipal, 576104 India; 4 Computer Engineering Department, Umm Al-Qura University, Mecca, 24381 Saudi Arabia

**Keywords:** 2-bit encoding, Nanomaterials, Aqueous, Machine learning, Food Safety, Graphene, Graphene, Nanobiotechnology, Nanoscale devices, Optical materials and structures, Optical techniques, Health care, Materials science

## Abstract

This investigation presents the development and characterization of an advanced piezoelectric perovskite-based biosensing platform optimized for formalin detection in aqueous media through the implementation of Locally Weighted Linear Regression (LWLR) machine learning algorithms. The sensor architecture operates within the terahertz spectral region and incorporates an advanced nanomaterial composite system comprising black phosphorus, gold nanostructures, graphene, and barium titanate to maximize detection sensitivity and operational performance metrics. The engineered platform integrates a circular graphene metasurfaces configuration with a gold-based H-resonator assembly and concentrically arranged circular ring resonators. Computational simulations demonstrate vigorous sensing capabilities across three discrete frequency bands, achieving remarkable sensitivity parameters of 444 GHzRIU⁻¹, accompanied by a quality factor of 5.970 and detection accuracy of 7.576. The integration of LWLR-based optimization protocols substantially enhances prediction accuracy while reducing computational time by ≥ 85% as well as cutting down the required resources. The proposed sensor architecture presents significant potential for environmental monitoring and clinical applications, offering a highly sensitive and efficient methodology for quantitative formalin detection in aqueous environments.

## Introduction

The detection of harmful chemicals in the environment and food supply is a critical challenge in safeguarding public health and safety. Among these chemicals, formaldehyde and its aqueous solution, formalin, stand out as particularly concerning contaminants^1^. Formaldehyde is a known carcinogen and exposure leads to various health issues ranging from respiratory problems to increased cancer risk^2^. Its widespread use in industrial processes, building materials and as a preservative makes it a ubiquitous threat that demands vigilant monitoring^3^. The IARC (2012) has listed formaldehyde as group 1 human carcinogen and its long-term exposure leads to nasopharyngeal cancer and myeloid leukaemia^4^. Employees exposed to formaldehyde have shown increased probability of contracting leukaemia than those who were not exposed and this probability increases with exposure level and time^5^.Research has also shown that workers in industries with high formaldehyde exposure exhibit an increased prevalence of respiratory symptoms and reduced lung function compared to those with lower exposure levels^6^.

Standard methods of detecting formalin include; ion chromatography (IC)^7^, high performance liquid chromatography (HPLC)^8^ among others. These methods have proved to be efficient over other conventional techniques. Ion chromatography is a technique that differentiates charged particles and polar compounds according to their electrical properties; nevertheless, it is generally tedious and time-consuming meaning that it cannot work efficiently in high throughputs^9^. Polarography, directly measures the current that flows in a solution as a function of applied voltage; this method is specific and sensitive for detecting formalin; however, it possesses some problems such as method complexity, need for highly purified reagents and constant check of the equipment^10^. Gas chromatography is very efficient particularly for volatile organic compounds; its resolution and sensitivity are usually very high, but GC systems are usually very costly and require frequent maintenance, not to mention that most of the time the samples for analysis require very thorough preparation^11^. Spectrophotometric method is simpler and more accessible than other methods, however, it is characterized by somewhat lower sensitivity and specificity in comparison with others^12^. Technological advancement in Chromatographic methodology also contributes to some inaccuracies because other substances in the sample also affect the result making it less accurate in matrices of different complexity.

In recent years, there has been increasing interest in creating fast, on-site methods for detecting formaldehyde and formalin. Biosensors, in particular, have shown great promise because they can be miniaturized, provide real-time results, and are highly sensitive^13^. Different types of sensing platforms, such as electrochemical, optical, and piezoelectric sensors, have been investigated. Among these, optical sensors that utilize surface plasmon resonance (SPR) stand out due to their ability to detect without labels and their high sensitivity to changes in the refractive index^14–17^.

Surface plasmon resonance (SPR) is used to improve the sensitivity of the sensor^18^. SPR based sensors have demonstrated high sensitivity among other performance parameters which are used to improve the results^19^. SPR offers a rapid, highly sensitive, and selective method for real-time monitoring of molecular interactions without the need for labelling. The key strength of the method is the capability to offer quantitative data on molecular interactions that is helpful at the stage of drug discovery among other biosensing applications^20^. It enables the investigation of different kinds of biomolecular complexes such as protein-protein, protein-DNA, and protein-lipid^21^.Recent advancements in plasmonic sensors involve the application of nanostructured surfaces and metamaterials to boost sensitivity and broaden the spectrum of detectable analytes^22,23^.

Other developments in photonic biosensing have also been realized from the improvements in light sources and detectors^24^. Thanks to the advancements in material science accompanied by the superior miniaturization of compact high-performance lasers and effect photodetectors. The portable biosensing devices have emerged, which are paving way to the actual portable diagnostically advanced devices that may be used at the point of care^25^.

The inclusion of artificial intelligence and machine learning techniques has boosted photonic biosensors’ data analysis features tremendously. Such computational strategies enhances the performance of signal interpretation of multiple sensors and analyte differentiation^26^.

Progress in nanotechnology and materials science has created new opportunities for improving sensor performance. Innovative materials like graphene, black phosphorus, and metasurfaces have demonstrated potential in boosting the sensitivity, selectivity, and response time of sensors^27^. When these materials are incorporated into thoughtfully designed structures, they display remarkable optical and electronic properties that enhance the sensor’s ability to detect even subtle changes in the environment^28,29^. Nanomaterials including black phosphorus, gold, graphene, perovskites like barium titanate (BaTiO_3_) greatly enhance the SPR sensor because of their unique characteristics. Black phosphorus(Bp) has higher anisotropy and tunable bandgap, making the sensitivity even higher^30^. On the other hand, gold is widely used in biosensing applications because of its highly stable and strong plasmonic response^31,32^. Graphene provides a number of advantages such as high electronic conductivity and specific surface area that increases the sensitivity and functionalization of SPR^33^.

In the present work, an SPR based sensor is proposed for formalin detection in water based on the integration of advanced nanomaterials and enhanced metasurfaces geometry. The proposed sensor is designed in the terahertz regime. The integration of Bp, Au, graphene, and perovskites such as barium titanate (BaTiO_3_) are designed to enhance the sensor’s sensitivity among other parameters. The features like tunable bandgap and large surface area of black phosphorus as well as strong electronic conductivity and a large surface area of graphene improves the performance of the sensor. Using these advanced materials with the metasurfaces point of view, the SPR sensor which is proposed here results in a satisfactory technique to detect formalin in water for environmental monitoring and health care applications.

## Design and modelling

**Fig. 1:**
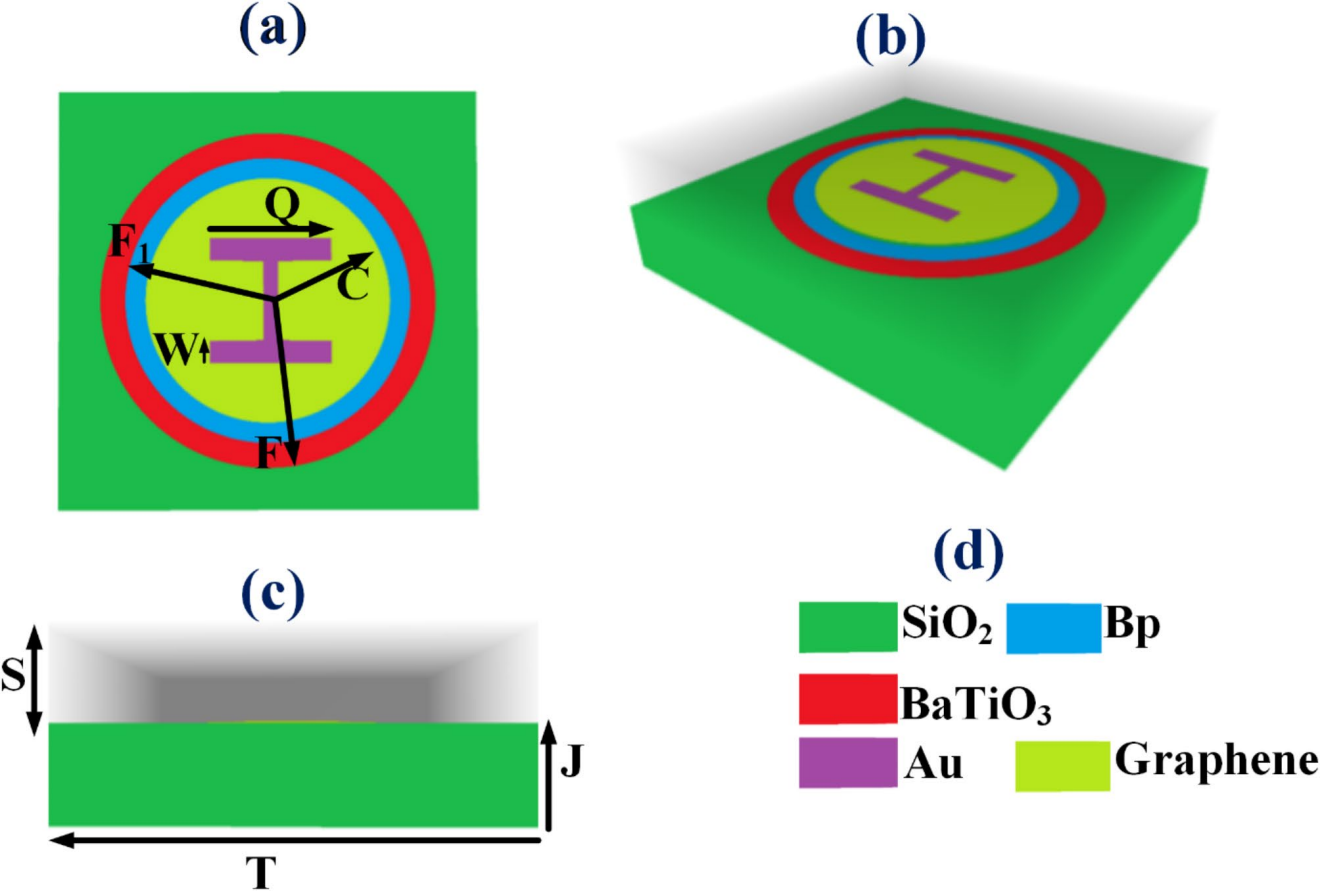
Proposed refractive index sensor for formalin detection: (**a**) top view, (**b**) 3D view, and (**c**) front view of the design and (**d**) depicts the design materials used in the sensor. Key parameters include: structure length and width, T = 20 μm for the structure’s thickness, a SiO_2_ layer with a thickness of J = 1 μm, analyte thickness S = 4 μm, graphene metasurfaces layer height set to 0.34 nm, and a central circle radius C = 4.3 μm.

The proposed formalin detection sensor’s schematic design is illustrated in Fig. 1a to d, which presents multiple structural perspectives, including 3D, overhead, and frontal views. The sensor’s modelling process begins with the careful placement of a SiO_2_ substrate, chosen for its excellent dielectric properties and compatibility with nanoscale structures. Upon this foundation, a circular graphene metasurface pattern is precisely superimposed, Subsequently, a gold-based H-resonator is constructed atop the circular resonator. This H-shaped structure is then coated with gold, selected for its superior conductivity and plasmonic properties, which are crucial for the sensor’s functionality. Two concentric circular ring resonators encompass these central structures, creating a layered sensing mechanism. The outer ring is coated with BaTiO_3_, a ferroelectric material renowned for its high dielectric constant and superior piezoelectric characteristics. The inner ring, in contrast, is coated with black phosphorus, The device’s key dimensional parameters are precisely calibrated to optimize performance: the overall structure length and width measure 20 μm (T). The SiO_2_ substrate thickness is set at 1 μm (J). The analyte layer, crucial for formalin interaction, has a thickness of 4 μm(S). The graphene metasurface layer height is set at 0.34 nm. The central circle, forming the core of the sensing area, has a radius of 4.3 μm(C), while the inner and outer circular rings have radii of 5.6 μm (F_1_) and 6.2 μm (F), respectively. These dimensions are optimized to create resonant conditions that enhance the sensor’s response to formalin. The H-shaped resonator, a critical component for electromagnetic field concentration, measures 6 μm in length(Q) and 0.5 μm in width(W). The sensor operates on principles of plasmonic resonance and electric field enhancement, where the presence of formalin molecules alters the local electromagnetic environment, leading to detectable changes in the sensor’s response. Overview of the sensor parameters given in Table 1.

The refractive index of silicon dioxide (SiO₂) is expressed in different ways depending on the wavelength of light being used. One common empirical formula used to describe the refractive index SiO_2_ is^34^;

1 $$\:n\left(\lambda\:\right)=\sqrt{1+\frac{{B}_{1}C}{{\lambda\:}^{2}-{C}_{1}}+\frac{{B}_{2}{\lambda\:}^{2}}{{\lambda\:}^{2}-{C}_{2}}+\frac{{B}_{3}{\lambda\:}^{2}}{{\lambda\:}^{2}-{C}_{3}}}$$

The Drude model is a classical approach used to describe the electrical and optical properties of metals by treating free electrons as a gas of non-interacting particles. For the relative permittivity (or dielectric function) of gold, the Drude model provides a framework for understanding how the metal’s permittivity changes with frequency. This permittivity is given by^37^;

2 $$\:{\varepsilon\:}_{Au}={e}_{8}-\frac{{\omega\:}_{D}^{2}}{\omega\:\left(\omega\:+j{\gamma\:}_{D}\right)}\:-\:\frac{{{\Delta\:}}_{\varepsilon\:}{{\Omega\:}}_{L}^{2}}{\left({\Omega}^{2}-{{\Omega\:}}_{L}^{2}\right)+j{{\Gamma\:}}_{L}\omega\:}$$

**Table 1:** Overview of the sensor design aspects.

Parameter	T	J	S	F_1_	F	C	W	Q
Length(µm)	20	1	4	5.6	6.2	4.3	0.5	6

### Fabrication feasibility

**Fig. 2:**
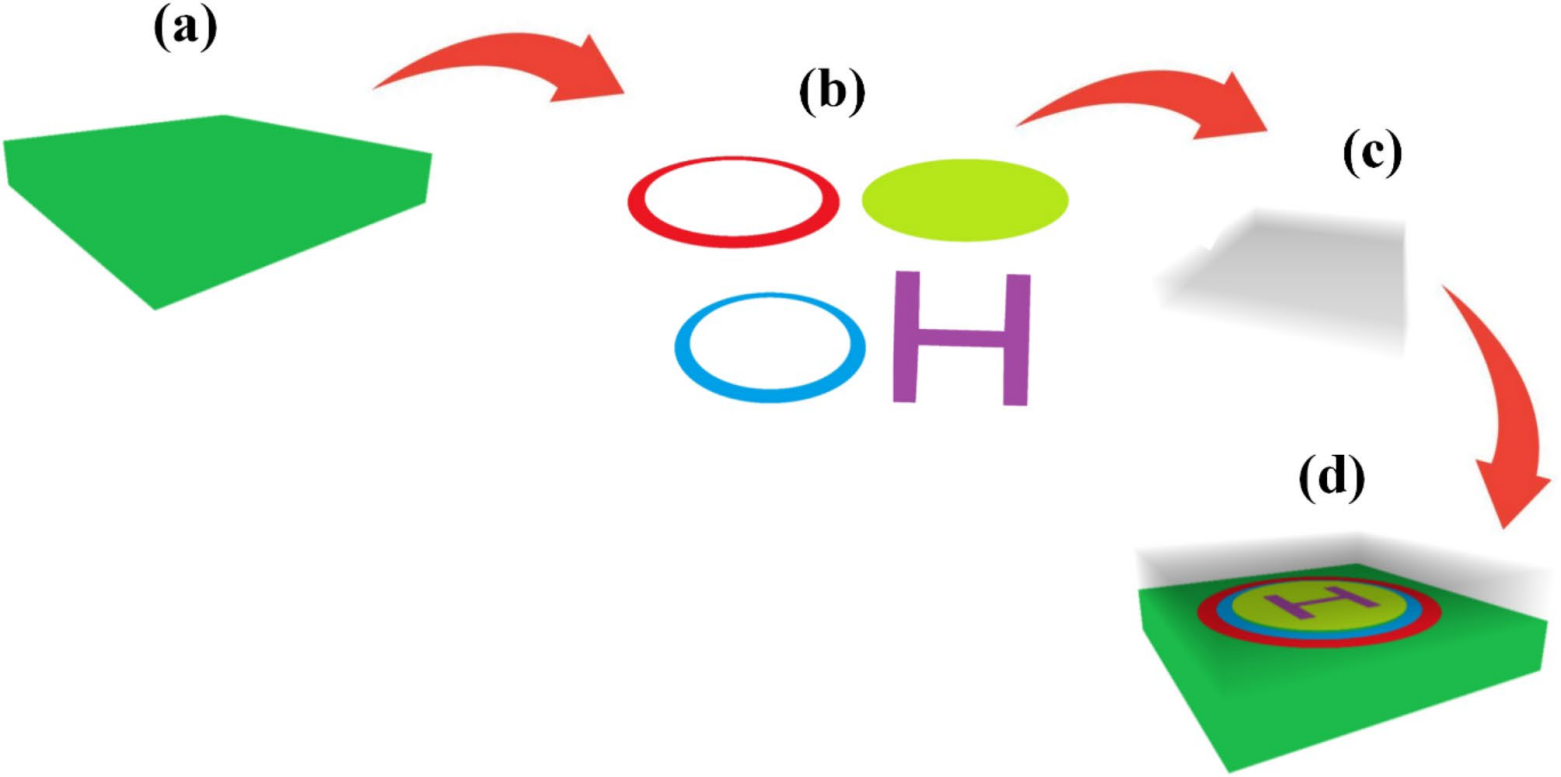
Proposed sensor fabrication process.

The fabrication process of the proposed sensor as shown in Fig. 2 in real time laboratories starts with a silicon wafer upon which an exactly required thickness of silicon dioxide is thermally grown. Real-time ellipsometry allows the feedback while building this layer, to verify the thickness of this layer to meet with the particular one micron required. The second step presents the graphene metasurface which is one of key elements of the sensor. A single layer of graphene is carefully transferred or grown onto the silicon dioxide substrate, with Raman spectroscopy. The circular pattern is then ‘printed’ onto the graphene using state of the art lithography and the process is carefully controlled to result in the required 4.3 μm radius. The Au based H-resonator is manufactured through the deposition process and the further patterning. Because gold is deposited through accurate evaporation, a quartz crystal microbalance gives thickness information in real-time. The wedge structure, having a H-shape, is then chemically eroded or engraved and frequent optical examination is done to ensure the best results. The concentric circular ring resonators are produced next with each of them made from a different chemical composition. Thus, the outer ring, with its 6.2 μm radius is made of barium titanate and the inner 5 μm layer is made of Bp. Employing black. Physical deposition includes methods like a pulsed laser deposition or chemical vapor deposition; in-situ spectroscopic ellipsometry used to feeding information about the thickness and quality of the films. The last structure layer – a 4 μm thick analyte layer intended for the interaction with formalin – is deposited and its thickness is strictly controlled using the real-time profilometry. As each component of the sensor takes shape, a battery of real-time tests is conducted to verify its properties and performance. Spectroscopic ellipsometry and reflectance measurements give the immediate optical characterization of the structure to study the plasmonic behaviour. Electrical properties are checked in the real time manner by passing current and voltage through the circuit to verify that the graphene and metal layers are switching as they are designed to. The structural integrity and the high precision of the put forth design is evidenced through the in-situ scanning electron microscopy and atomic force microscopy of the sensor for the visualization of its nano features. These techniques are applied on the flow to meet the required standard for the corresponding component in fabrication. To evaluate the sensor’s ability to detect formalin, the microfluidic apparatus is used with actual formalin samples. This setup enables the formalin solution to be applied to the sensor surface in a controlled way; surface plasmon resonance spectroscopy permits real time monitoring of the sensors. At the same time, alterations in the electrical resistance of a layer created from graphene are recorded, thus giving a kind of a differential perspective on how the sensor works. Integration of performance appraisal includes exposure of the sensor to different concentration of formalin, other chemicals in order to test its selectivity. Sophisticated data acquisition software gathers and processes these outcomes in real time and, thus, enables the user to determine the sensor’s sensitivity and specificity at once. Finally, the sensor is subjected to environmental stress tests. It is also tested for its performance over time and under different temperatures and humidity level to check its status of function and detection.

### Metasurfaces analysis

Graphene’s remarkable conductivity has important effects across multiple fields. In electronics, its superior mobility and conductivity position it as a prime material for next-generation transistors, sensors, and fast communication components^35–39^. In materials science, graphene is employed to develop conductive coatings, composites, and flexible electronic devices. The conductivity σs of graphene is derived from Eq. 3 to 6^40–42^.

3 $$\:\varepsilon\:\:\left(\omega\:\right)=1+\:\frac{{\sigma\:}_{s}}{{\varepsilon\:}_{0}\omega\triangledown\:}$$

4 $$\:{\sigma\:}_{intra}=\:\frac{-j{e}^{2}{k}_{B}T}{\pi\:{\hslash\:}^{2}\:(\omega\:-j2{\Gamma})}\left(\frac{{\mu\:}_{c}}{{k}_{B}T}+2\:\text{ln}\left({e}^{\frac{{\mu\:}_{c}}{{k}_{B}T}}+1\right)\right)$$

5 $$\:{\sigma\:}_{inetr}=\:\frac{-j{e}^{2}}{4\pi{\hslash\:}\:}\text{ln}\left(\frac{2\left|{\mu\:}_{c}\right|-\left(\omega\:-j2{\Gamma}\right){\hslash\:}}{2\left|{\mu\:}_{c}\right|+\left(\omega\:-j2{\Gamma}\right){\hslash\:}}\right)\:$$

6 $$\:{s}_{s}={s}_{intra}+{s}_{inter}$$

Metasurfaces are engineered materials with periodic structures designed to manipulate electromagnetic waves in novel ways^43^. Their effective permeability and permittivity are derived from the material’s structure and its interaction with electromagnetic fields. The equations for these properties are often presented in specific forms based on the underlying theoretical models. Some of these equations are^44^;

7 $$\:Z=\pm\:\sqrt{\frac{{\left(1+{s}_{11}\right)}^{2}-{s}_{21}^{2}}{{\left(1-{s}_{11}\right)}^{2}-{s}_{21}^{2}}}$$

8 $$\:\:{e}^{in{k}_{0}d}=\frac{{s}_{11}}{1-{s}_{11}\frac{2-1}{2+1}}$$

9 $$\:n=\frac{1}{{k}_{0}d}\left\{\right[ln{e}^{in{k}_{0}d}\left)\right]{\prime\:}{\prime\:}+2mp\}-i[\text{l}\text{n}\left({e}^{in{k}_{0}d}\right)\left]{\prime\:}\right]$$

10 $$\:\epsilon\:=\frac{n}{z}$$

11 $$\:\mu\:=nz$$

12 $$\:A\left(w\right)=1-R\left(w\right)-T\left(w\right)=1-{\left|{s}_{11}\right|}^{2}-{\left|{s}_{21}\right|}^{2}$$

### Detection of Formalin leveraging RIs

Refractive index is the ratio of the speed of light in a vacuum to that in the substance or material being referred to. It is defined as total relative average speed of the substance, which is constructed as the reciprocal of the refractive index of the substance with respect to the speed of light in vacuum. Actually, different substances bend the light differently, therefore, finding the refractive index determines and identify the concentrations of the existing substances. Even if a small amount of formalin is mixed with water the solution’s entire refractive index changes due to the fact that the refractive index of formaldehyde is different than water. If one wanted to determine the presence of formalin one would begin by making a sample of the water, which contains formalin, ensure that the sample is mixed well and has no containments that could skew the result. The reading is then obtained using a calibrated instrument – refractometer-which is specially made to measure refractive index of a solution. This device determines the amount of light deviation as the light is passed through the particular sample. Calibration is crucial here: in order to assay on the refractometer, they have to put it through standard solutions of known RI to calibrate it. After one sets the refractometer, it is possible to have a value of the refractive index of the sample being analysed. Refractive index of water is approximately 1 and for the pure water it is close to 1.333, and with introduction of the formalin the refractive index raises in dependence with the concentration of the formaldehyde. Table 2 demonstrates the RIs at different concentrations of formalin.

**Table 2:** RI(RIU) for Formalin detection^45^.

Category	*n*_1_	*n*_2_	*n*_3_	*n*_4_	*n*_5_
n	1.33	1.3345	1.339	1.348	1.36
Concentration(nM)	00	25	50	100	200

## Results and discussion

The design presented in Fig. 1 undergoes extensive simulation using COMSOL Multiphysics version 6.2. The simulation yields various results, including transmittance plots, electric field intensity distributions, and a comprehensive sensitivity analysis based on Figure of Merit and Q-factor. In the quest for optimal formalin detection, a systematic parametric study was done; several key structural elements of the design, including the radii of the circular rings and central circle, the dimensions of the H-shaped resonators, the graphene chemical potential and the angle of incident light were optimized. This optimization process aims to identify the configuration that exhibits the highest sensitivity to formalin presence. A series of figures presented in this section demonstrates how structural modifications influence the transmittance characteristics of the sensor design. Each figure corresponds to a specific parameter variation, illustrating the effects of adjusting elements such as ring radii, central circle dimensions, H-resonator geometry, graphene properties, and incident light angles.

### Optimization of Graphene chemical potential (GCP) and angle of incidence

**Fig. 3:**
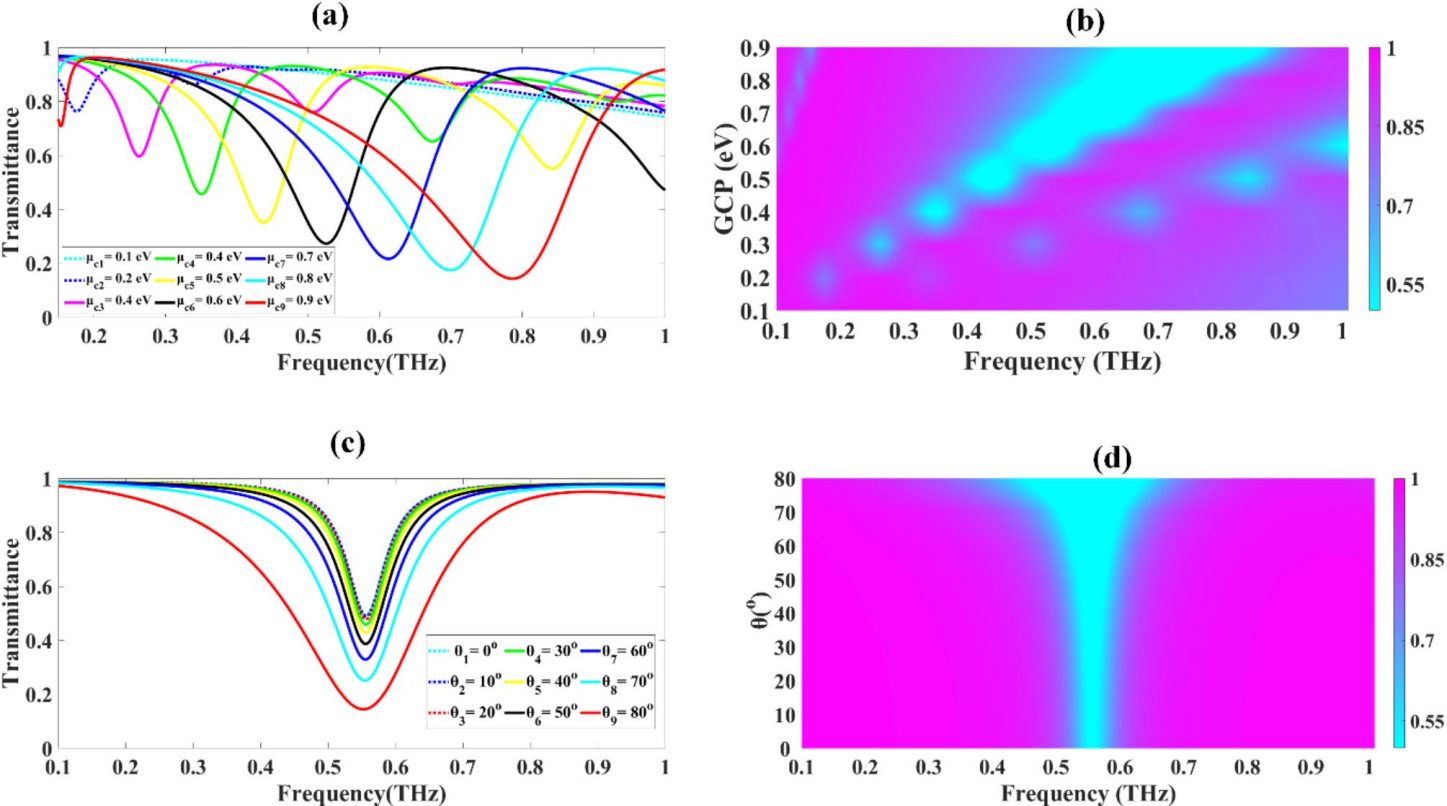
Demonstrates the sensor’s transmittance spectrum shifting towards higher frequencies with increasing GCP (**a**-**b**), while maintaining angle-independent spectral features across varying incidence angles (**c**-**d**).

The GCP was methodically adjusted across a range of 0.1 to 0.9 electron volts (eV), with the outcomes displayed in Fig. 3a and b. When the GCP was set to lower values (between 0.1 and 0.2 eV), the transmittance remains largely unchanged within 0.1-1THz. However, as the GCP is incrementally raised from 0.3 to 0.8 eV, we observe a progressive reduction in transmittance.

The sensor’s ability to transmit terahertz radiation decreases as the GCP increases. When the GCP is set to 0.3 eV, the transmittance is 74%. A slight increase to 0.4 eV GCP results in a transmittance of about 76% at the same frequency. The trend of decreasing transmittance continues with higher GCP values. At 0.5 eV GCP, transmittance drops to 60% near 0.26 THz, while with 0.6 eV GCP, it decreases further to about 46% around 0.35 THz. As the GCP is raised to 0.7 eV, transmittance shifts to 35% at 0.44 THz. At 0.8 eV GCP, the transmittance drops to 27% near 0.53 THz. This pattern clearly demonstrates the inverse relationship between GCP and transmittance across the tested frequency range. The lowest transmittance reduction of 21.69% is observed at a resonance frequency of 0.612 THz for a GCP of 0.9 eV, as depicted in Fig. 3a. Figure 3b presents a Fermi plot that clearly demonstrates the correlation between increasing GCP and both the blue shift in transmittance and the progressive increase in transmittance drop. Based on these results, a GCP value of 0.9 eV was selected to optimize sensitivity and maximize transmittance drop. When the chemical potential changes, the density of states near the Fermi level also changes, altering how electrons interact with incoming electromagnetic waves. This shift in electron occupation increases the probability of specific electronic transitions, leading to higher absorption and lower transmittance. Additionally, the chemical potential influences the photon frequencies coupled to plasmon resonances in graphene with high intensity. Plasmons are vibrations of the free electrons in the material induced by the electromagnetic field; their resonant frequencies vary with the chemical potential. When these resonances align with the frequency of the incident light, absorption increases, resulting in reduced transmittance. Figure 3c and d further demonstrate the impact of the incident angle on the transmittance characteristics of the proposed sensor. Figure 3c and d present the findings from a comprehensive assessment of how the angle of incidence influences the transmittance curve. This analysis was conducted to determine whether the transmittance reaches its maximum value at any particular incidence angle. Data were collected for incidence angles ranging from 0° to 80°, with measurements taken at 10° intervals. Figure 3c shows that the transmittance plot exhibits minimal variation as the angle of incidence changes from 0° to 50°. Within this range, only a slight decrease in transmittance is observed, indicating that the proposed RIS maintains relatively consistent performance across these angles. This stability suggests that the sensor is largely insensitive to changes in incidence angle within this range, a desirable characteristic for practical applications. However, the behaviour becomes more complex at higher angles and frequencies. As illustrated in Fig. 3d, beyond the resonance frequency, the rate at which maximum transmittance is achieved diminishes. A detailed analysis of the transmittance drops across the full range of measured angles reveals a gradual decrease in transmittance drop as the angle of incidence increases. At 0°, a transmittance drop of 49.40% is attained at 0.556 THz. This value remains relatively stable up to 30°, with only a slight variation to 45.87%. However, as the angle increases further, the transmittance drop becomes more pronounced. At 50°, it shifts to 38.69%, still at 0.556 THz. For angles above 50°, not only does the transmittance drop continue to decrease, but there is also a slight shift in the frequency at which the maximum drop occurs. At 60°, the drop is 32.95% at 0.555 THz, at 70° it’s 25.16% at 0.554 THz, and at the extreme angle of 80°, the drop is 14.53% at 0.552 THz.

### **Optimization of resonators**

**Fig. 4:**
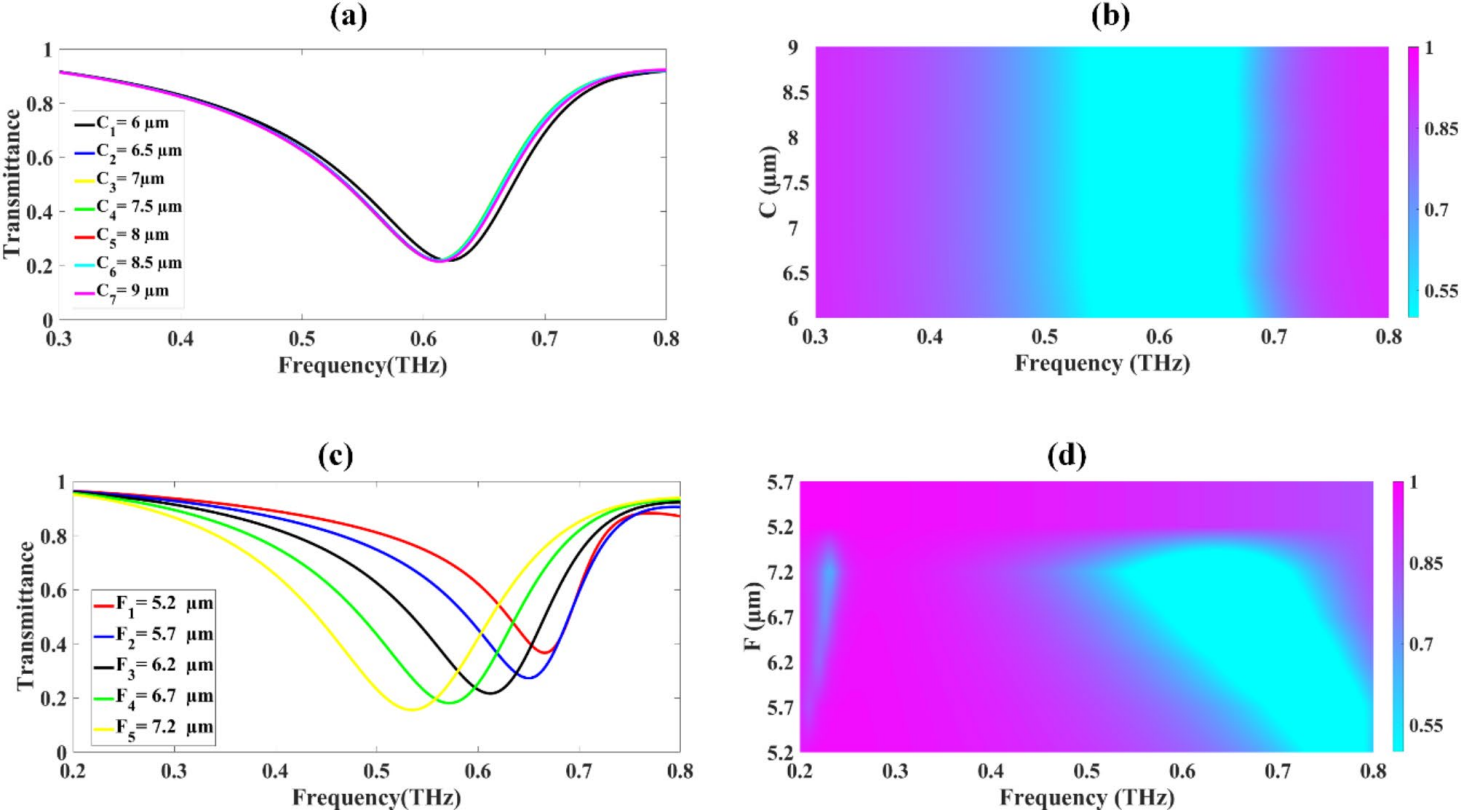
Illustrates the transmittance response resulting from the variations in both the circular resonators (**a**, **b**) and the circular ring resonators (**c**, **d**).

The impact of the circular resonator’s size on the sensor’s transmission properties is depicted in Fig. 4a and b. These graphs present data for frequencies spanning from 0.1 to 0.8 terahertz. In this analysis, the radius of the circular resonator was systematically modified, beginning at 6 μm and gradually increasing to 9 μm. Each adjustment to the radius was made in small increments of 0.5 μm, allowing for a detailed examination of how this parameter influences the sensor’s performance across the studied frequency range.

Figure 4(a) demonstrates that altering the radius of the circular resonator has a relatively minor influence on the transmittance profile. A detailed examination of the transmittance drops for each radius value shows a consistent pattern: At 6.0 μm radius, a 21.875% drop occurs at 0.62 THz. For 6.5 μm, the drop is 22.001% at 0.612 THz. At 7.0 μm, a 21.694% drop is observed at 0.612 THz. For 7.5 μm, the drop is 21.946% at 0.61 THz. At 8.0 μm, a 21.663% drop occurs at 0.613 THz. For 8.5 μm, the drop is 21.883% at 0.612 THz. At 9.0 μm, a 21.557% drop is observed at 0.612 THz. These data points demonstrate that the transmittance drop remains remarkably stable, hovering around 21–22% across all tested radii. Furthermore, the frequency at which the maximum drop occurs stays consistently close to 0.612 THz, with only minor fluctuations. Figure 4(b) presents these results in the form of a colour plot, providing a visual representation of the transmittance behaviour across the range of radii and frequencies. This graphical depiction reinforces the observations from Fig. 4(a), clearly showing the consistency in transmittance characteristics despite changes in the resonator’s radius. The stability of both the transmittance drop magnitude and the frequency of maximum response across different resonator radii indicates that the sensor’s performance is not highly sensitive to small variations in this particular geometric parameter. Figure 4c and its accompanying Fermi plot in Fig. 4d demonstrates the effects of varying the dimensions of the two circular ring resonators on the transmittance response of the proposed sensor design. The analysis explores a range of resonator dimensions from 5.2 μm to 7.2 μm, with measurements taken at 0.5 μm intervals. This systematic variation depicts a significant impact on the sensor’s transmittance characteristics. As demonstrated in Fig. 4c, the transmittance drops exhibit a clear trend as the dimensions of the ring resonators increase: At 5.2 μm, a substantial drop of 36.693% is observed at 0.666 THz. For 5.7 μm, the drop decreases to 27.350% at a slightly lower frequency of 0.65 THz. At 6.2 μm, the drop further reduces to 21.694% at 0.612 THz. For 6.7 μm, the trend continues with an 18.134% drop at 0.572 THz. At 7.2 μm, the drop diminishes to 15.608% at 0.535 THz. For the 6.7 μm and 7.2 μm, a shift occurs with transmittance drops of 72.796% observed at 1 THz for both cases. The Fermi plot in Fig. 4d depicts the same findings, clearly illustrating the shift in transmittance response towards lower frequencies as the dimensions of the two circular ring resonators increase. This shift in the resonance frequency is a key characteristic of the sensor’s behaviour under these geometric variations. These results demonstrate that the dimensions of the circular ring resonators play a crucial role in determining the sensor’s transmittance properties.

**Fig. 5:**
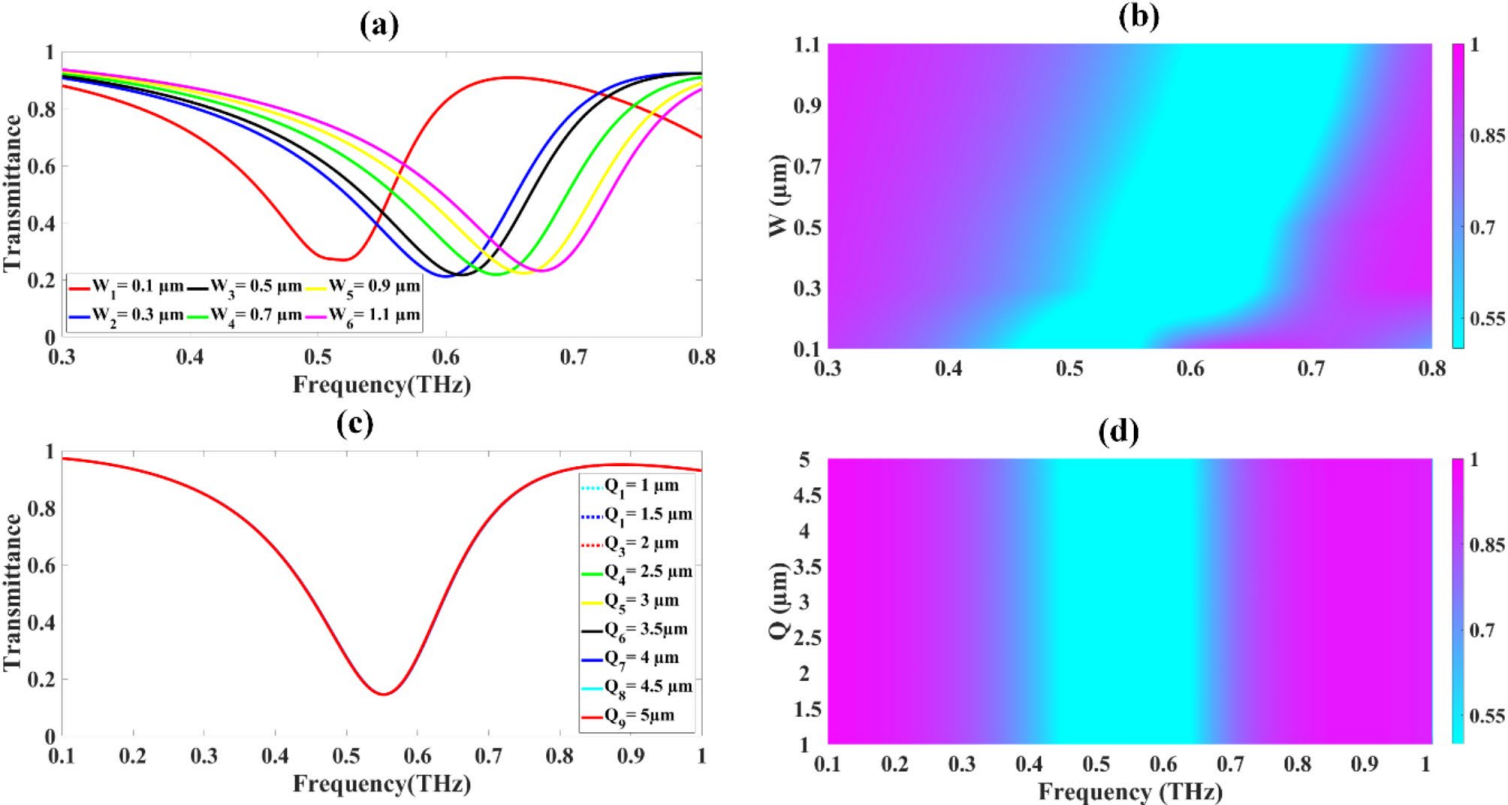
llustrates the transmittance response resulting from the variations in both the width (**a**, **b**) and the length (**c**, **d**) of the H shaped resonator.

Figure 5a-d presents a comprehensive analysis of how variations in the H-shaped resonator’s dimensions affect the transmittance response of the proposed sensor. This evaluation focuses on two key parameters: the width and length of the H-shaped resonator. Examining first the effects of varying the width of the H-shaped resonator, as illustrated in Fig. 5(a) and 5(b), we observe clear trend as depicted. In this case the width was systematically varied from 0.1 μm to 1.1 μm, with the resulting transmittance curves displayed in Fig. 5(a) and presented in a Fermi plot format in Fig. 5(b). The analysis demonstrates a non-linear relationship between the width of the H-shaped resonator and the transmittance characteristics of the sensor. At the smallest width of 0.1 μm, we observe the most pronounced transmittance drop of 26.900% occurring at 0.52 THz. As the width increases, the transmittance drop initially decreases before gradually increasing again. For instance, at 0.3 μm width, the drop is 21.150% at 0.6 THz, while at 1.1 μm width, it increases to 23.114% at 0.675 THz. Notably, there is a clear blue shift in the resonance frequency as the width of the H-shaped resonator increases. The frequency at which the maximum transmittance drop occurs steadily rises from 0.52 THz at the smallest width to 0.675 THz at the largest width tested. The Fermi plot in Fig. 5(b) offers the same demonstration of this phenomenon. It clearly shows how the transmittance response shifts towards higher frequencies as the width of the resonator grows. This graphical representation effectively captures the relationship between resonator width and frequency response. Figure 5c and d focus on the impact of the H-shaped resonator’s length on transmittance characteristics. This investigation examines the sensor’s behaviour across a broad frequency spectrum, extending from 0.1 to 1 terahertz. To conduct this analysis, we altered the H-shaped resonator’s length from 1 μm and progressively increased it to 5 μm, making fine adjustments of 0.5 μm at each step. The results depicted in Fig. 5c and d exemplifies that increasing the length of the H-shaped resonator has no significant impact on the transmittance characteristics of the sensor.

### Detection of formalin

**Fig. 6:**
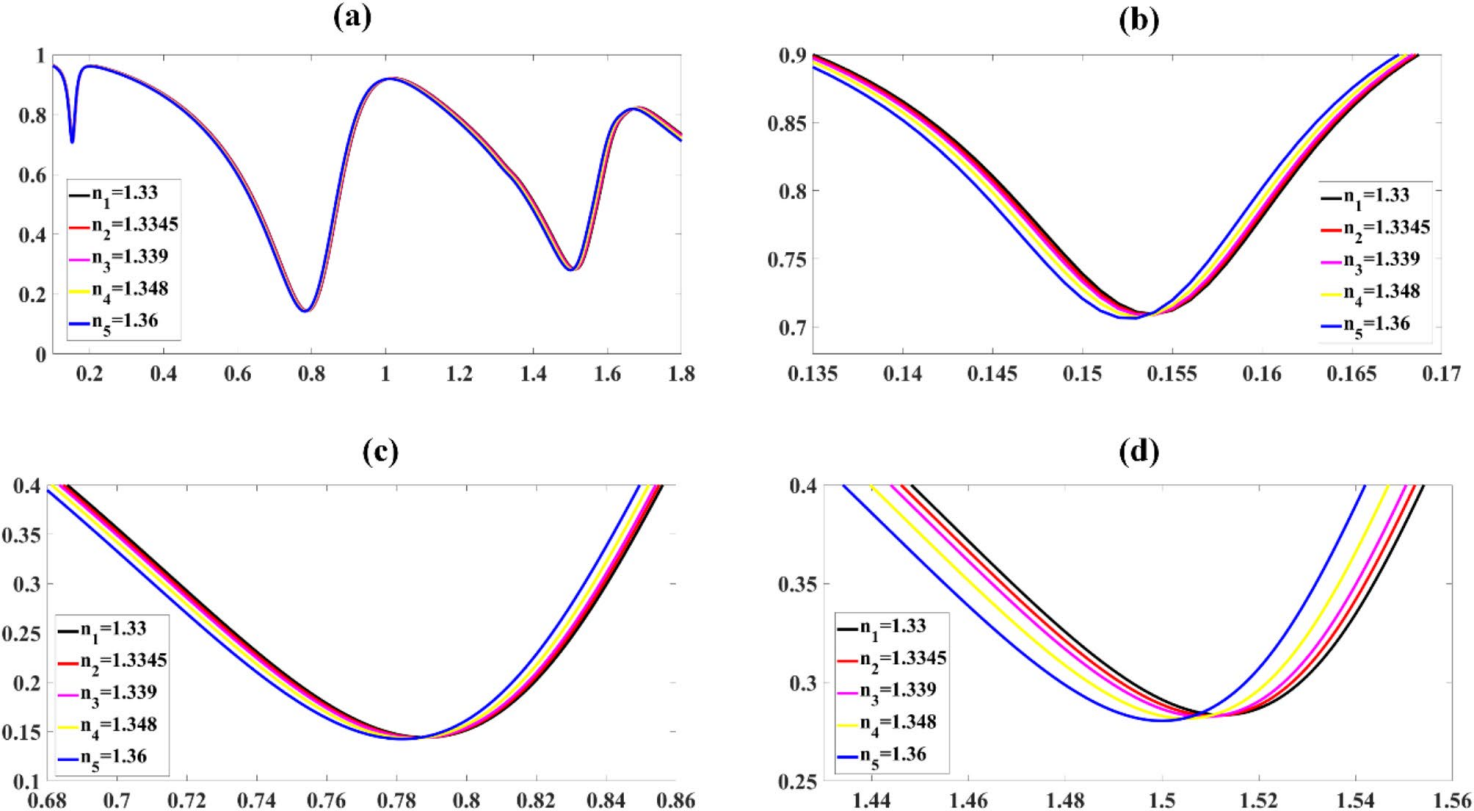
Presents formalin detection results, showing transmittance variations with RIs across three frequency bands (**a**), with detailed transmittance drop analysis for each band (**b**-**d**).

The formalin detection results are presented in Fig. 6. Figure 6(a) illustrates the spectral shift in the transmittance band as a function of varying formalin acid concentrations. This shift is indicative of the sensor’s response to different levels of formalin in the sample. Enhanced plots of the transmittance band shifts for three distinct frequency bands are provided in Fig. 6(b), (c), and (d).

**Fig. 7:**
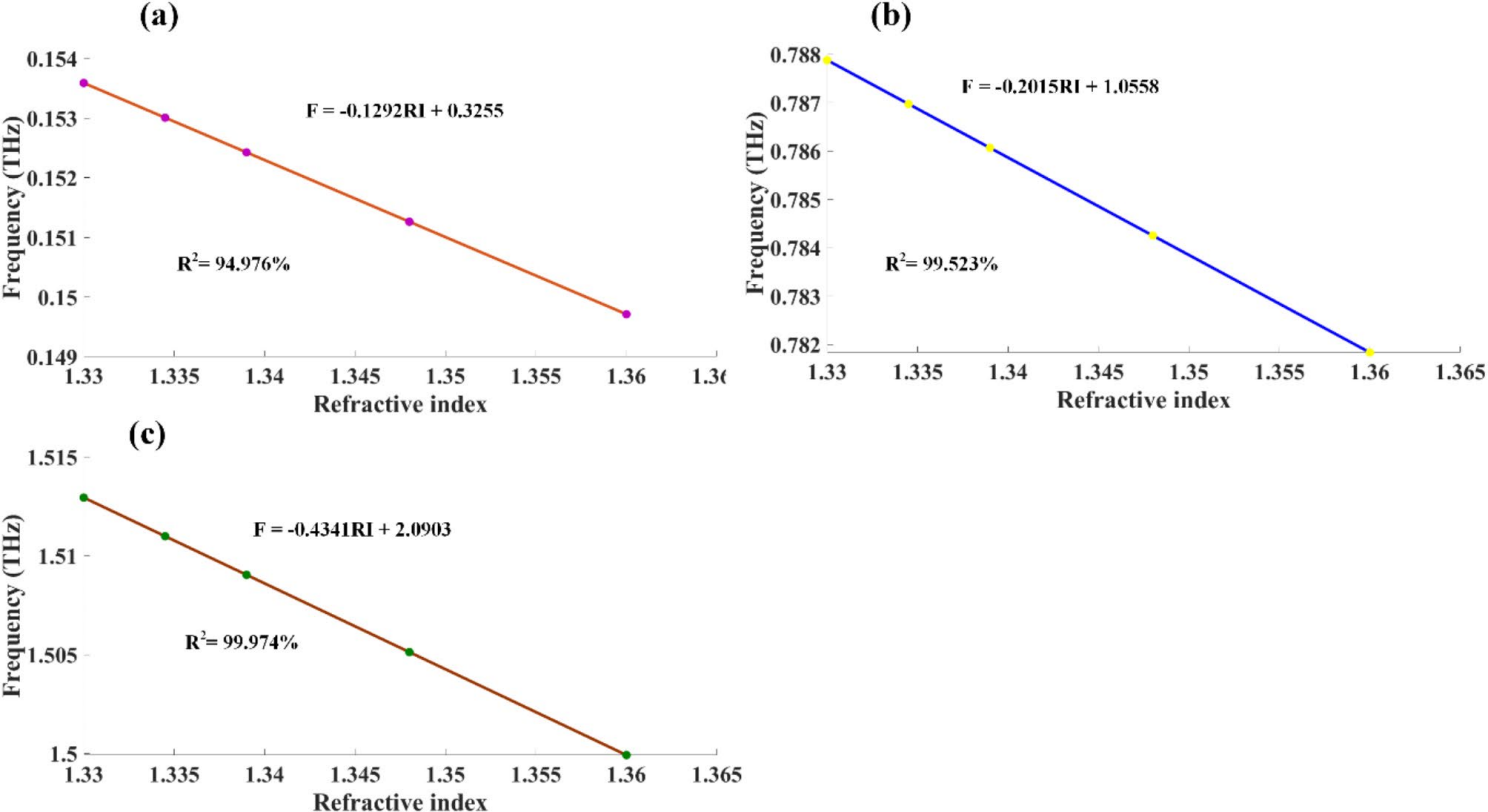
Shows curve fitting analysis demonstrating the relationship between resonance frequencies and refractive indices across three frequency ranges for formalin detection (**a**-**c**).

The spectral response is characterized by examining three distinct frequency bands. In the low-frequency band, depicted in Fig. 6b, minimum transmittance values were observed ranging from 70.920% at 0.154 THz to 70.631% at 0.150 THz. This band exhibits a relatively small change in transmittance of 0.289% across the concentration range, with a consistent leftward shift in the resonance frequency as formalin concentration increases. The mid-frequency band, shown in Fig. 6c, recorded minimum transmittance values from 14.413% at 0.788 THz to 14.262% at 0.782 THz. This band demonstrates a more pronounced change in transmittance of 0.151% compared to the low-frequency band, suggesting higher sensitivity to formalin concentration changes in this spectral region. In the high-frequency band, illustrated in Fig. 6d, transmittance minima were observed ranging from 28.346% at 1.513 THz to 28.049% at 1.500 THz. This band exhibits the largest change in transmittance of 0.297% and frequency shift of 13 GHz among the three bands, indicating the highest sensitivity to formalin concentration variations. The observed red-shift in resonance frequencies across all three bands with increasing formalin concentration is attributed to changes in the effective refractive index of the sensing medium. This shift is due to the interaction between formalin molecules and the sensor surface, altering the local dielectric environment. The varying degrees of sensitivity observed in different frequency bands suggest that the sensor’s response is frequency-dependent. The relationship between refractive indices (RIs) and resonance frequencies for three separate frequency bands is examined in Fig. 7a to c. The data reveals a strong linear correlation, which is mathematically expressed by Eq. 13 to 15 in the first frequency band, the relationship is described by:

13 $$\:\text{F}\:=\:-0.1292\text{R}\text{I}\hspace{0.17em}+\hspace{0.17em}0.3255$$

This equation has a remarkably high coefficient of determination (R²) of 94.976%, indicating an almost perfect linear relationship between RI and resonance frequency. For the second frequency band, the relationship is expressed as:

14 $$\:\text{F}\:=\:-0.2015\text{R}\text{I}\hspace{0.17em}+\hspace{0.17em}1.0558$$

This equation also shows a very strong linear correlation, with an R² value of 99.523%. For the second frequency band, the relationship is expressed as:

15 $$\:\text{F}\:=\:-0.4341\text{R}\text{I}\hspace{0.17em}+\hspace{0.17em}2.0903$$

This equation shows a very strong linear correlation, with an R² value of R^2^ = 99.974%.

The study also explored the correlation between the concentrations of analytes and their corresponding resonance frequencies. This analysis was conducted across three distinct frequency ranges, as illustrated in the graphical representations labelled 8a through 8d. To establish these relationships, a set of constants: K_1_, K_2_, and − 0.0001 are employed. Variable C denotes the concentration of the analyte under examination. Upon analysis, the findings demonstrate robust linear relationships within two of the examined frequency bands. For the initial frequency range, the correlation was mathematically expressed as follows:

16 $$\:\text{F}\:=\:-0.0000\text{K}1\text{C}\hspace{0.17em}+\hspace{0.17em}0.1534$$

This linear fit achieved a remarkably high coefficient of determination (R²) of 90.25%. For the second frequency band, the relationship is described by the equation:

17 $$\:\text{F}\:=\:-0.0000\text{K}2\text{C}\hspace{0.17em}+\hspace{0.17em}0.7877$$

This fit exhibits a high R² value of 96.983%.

For the third frequency band, the relationship is described by the equation:

18 $$\:\text{F}\:=\:-0.0001\text{C}\hspace{0.17em}+\hspace{0.17em}1.5125$$

**Fig. 8:**
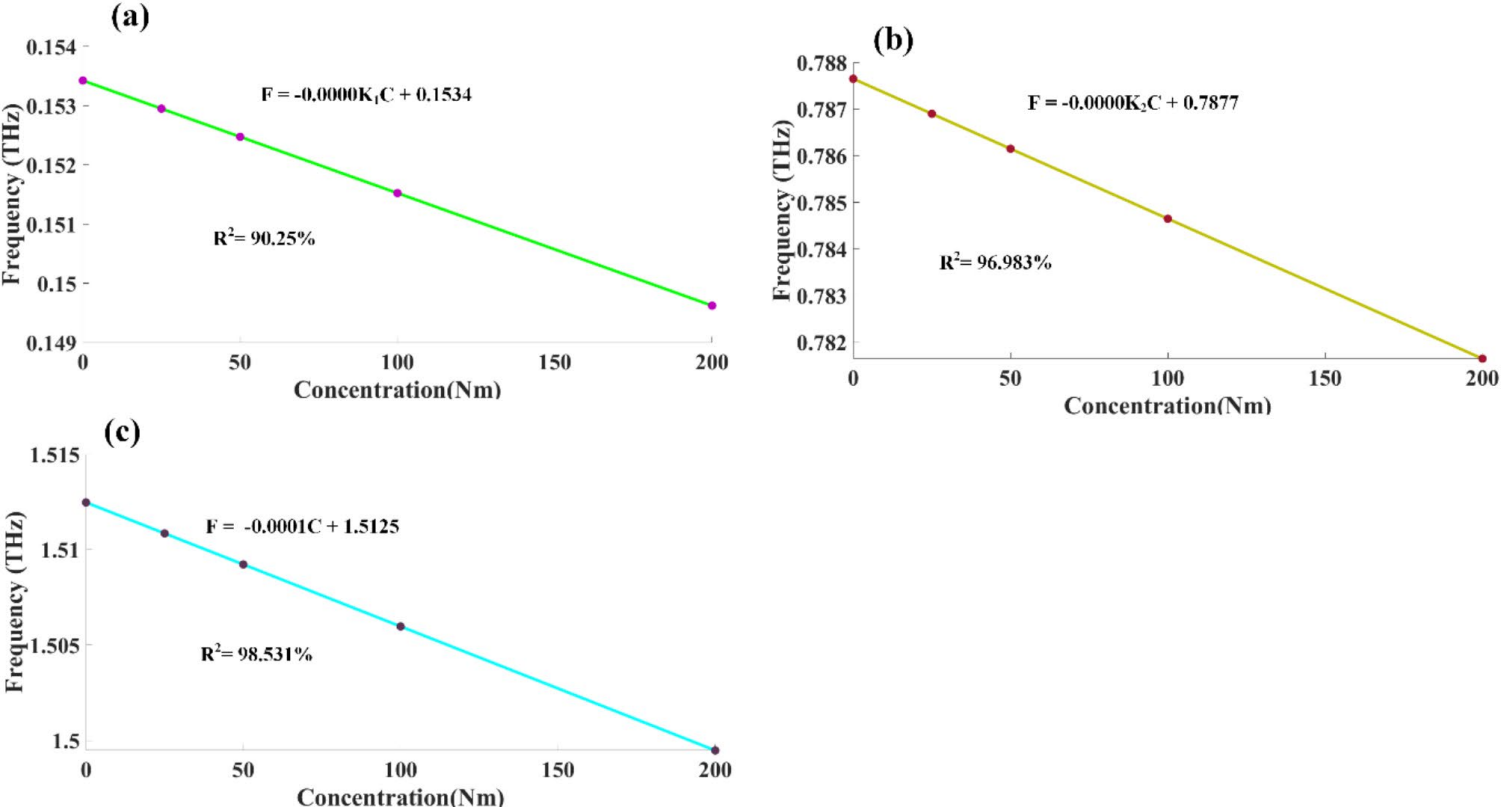
Displays curve fitting results showing the relationship between resonance frequencies and formalin concentrations across three frequency intervals (**a**-**c**).

This fit exhibits a high R^2^ = 98.531%. The relationship between refractive indices (RIs) and concentrations for three separate frequency bands is examined in Fig. 8a to c.

### Electric field intensity analysis

**Fig. 9:**
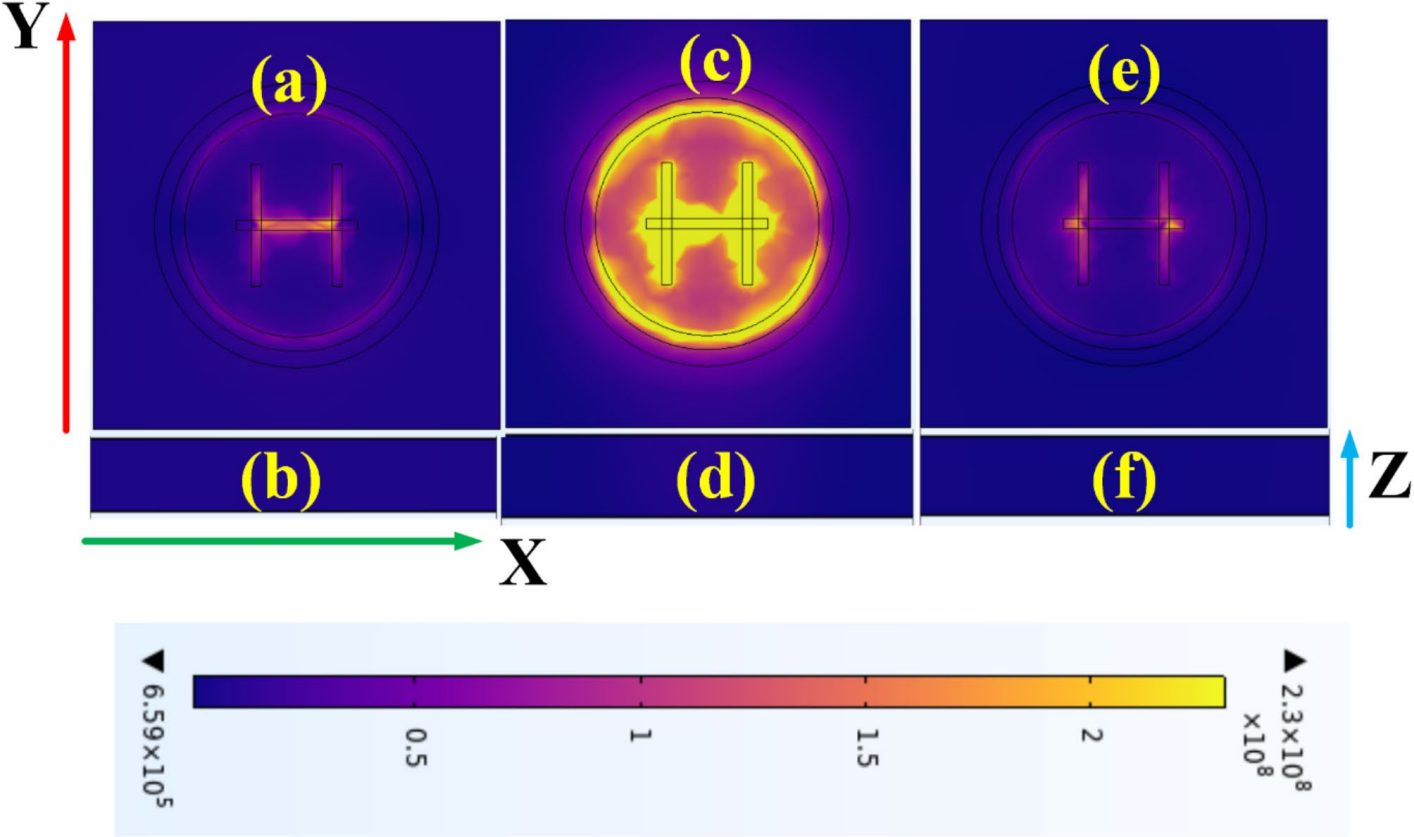
Electric field intensity for the proposed sensor at various frequencies: (**a**-**b**) 0.58 THz, (**c**-**d**) 0.4 THz, (e-f) 0.8THz, and (**d**) 1THz.

Figure 9 provides a detailed analysis of the electric field intensity (EFI) results for the proposed sensor, evaluating its performance within the 0.1 THz to 1.8 THz frequency range. The sensor’s behaviour is depicted at three specific frequency points: 0.4 THz, 0.8 THz, and 1.0 THz. The results demonstrate a clear trend in the sensor’s absorption and transmittance properties. At 0.8 THz, as demonstrated in Fig. 9c and d, there is a notable increase in absorption coupled with a corresponding decrease in transmittance. This phenomenon is particularly significant as it represents the sensor’s peak performance in terms of absorption. In contrast, the sensor’s behaviour at both lower and higher frequencies presents same results. At 0.1 THz, depicted in Fig. 9a and b, and at 1.8 THz, shown in Fig. 9e and f, the absorption decreases markedly. Simultaneously, the transmittance at these frequency extremes approaches near-unity, indicating that most of the incident electromagnetic energy passes through the sensor with minimal interaction. This varying response across the frequency range suggests a finely tuned resonance behaviour of the metasurface. The gradual increase in absorption as the frequency approaches 0.8 THz, followed by a subsequent decrease at higher frequencies, indicates a carefully designed frequency selectivity.

The EFI behaviour is attributed to the specific geometric parameters of the metasurface structure, possibly including the size, shape, and arrangement of its constituent elements.

There are many parameters that defines the performance of any sensor. Some of these parameters include;

19 $$\:\text{S}=\frac{\varDelta\:f}{\varDelta\:n}$$

20 $$\:\text{F}\text{O}\text{M}=\:\frac{S}{FWHM}$$

21 $$\:\text{Q}=\frac{fr}{FWHM}$$

22 $$\:\text{D}\text{L}=\:\left(\frac{\varDelta\:n}{1.5}\right)\times\:{\left(\frac{FWHM}{\varDelta\:f}\right)}^{1.25}$$

23 $$\:\text{D}\text{R}=\:\frac{{f}_{r}}{\sqrt{FWHM}}$$

24 $$\:\text{S}\text{N}\text{R}=\frac{{\Delta\:}f}{FWHM}$$

25 $$\:\text{S}\text{R}=\:S\:\times\:\:DL$$

26 $$\:\text{D}\text{A}=\:\frac{1}{FWHM}$$

27 $$\:\text{X}=\frac{2{\left({\Delta\:}f\right)}^{0.75}{\left(FWHM\right)}^{0.25}}{9}$$

The parameters of the proposed sensor across the three frequency bands calculated using the above formulas are presented in Tables 3, 4 and 5.

**Table 3:** Overview of sensor results for the first frequency band depicted in Fig. 6b.

f(THz)	0.154	0.153	0.152	0.151	0.15
n(RIU)	1.33	1.3345	1.339	1.348	1.36
Df(THz)		0.001	0.001	0.001	0.001
Dn(RIU)		0.0045	0.0045	0.009	0.012
S(GHzRIU^− 1^)		222	222	111	83
FWHM(THz)	0.276	0.276	0.276	0.276	0.276
FOM(RIU^− 1^)		0.805	0.805	0.403	0.302
Q	0.558	0.554	0.551	0.547	0.543
DL		0.012	0.012	0.024	0.033
DR	0.293	0.291	0.289	0.287	0.286
SNR		0.004	0.004	0.004	0.004
SR		0.003	0.003	0.003	0.003
DA	3.623	3.623	3.623	3.623	3.623
X		0.001	0.001	0.001	0.001

**Table 4:** Overview of sensor results for the first frequency band depicted in Fig. 6c.

f(THz)	0.788	0.787	0.786	0.784	0.782
n(RIU)	1.33	1.3345	1.339	1.348	1.36
df(THz)		0.001	0.001	0.002	0.002
Dn(RIU)		0.0045	0.0045	0.009	0.012
S(GHzRIU^− 1^)		222	222	222	167
FWHM(THz)	0.132	0.132	0.132	0.132	0.132
FOM(RIU^− 1^)		1.684	1.684	1.684	1.263
Q	5.970	5.962	5.955	5.939	5.924
DL		1.342	1.342	1.129	1.505
DR	2.169	2.166	2.163	2.158	2.152
SNR		0.008	0.008	0.015	0.015
SR		0.298	0.298	0.251	0.251
DA	7.576	7.576	7.576	7.576	7.576
X		0.001	0.001	0.001	0.001

**Table 5:** Overview of sensor results for the first frequency band depicted in Fig. 6d.

F(THz)	1.513	1.511	1.509	1.505	1.5
N(RIU)	1.33	1.3345	1.339	1.348	1.36
Df(THz)		0.002	0.002	0.004	0.005
Dn(RIU)		0.0045	0.0045	0.009	0.012
S(GHzRIU^− 1^)		444	444	444	417
FWHM(THz)	0.305	0.305	0.305	0.305	0.305
FOM(RIU^− 1^)		1.457	1.457	1.457	1.366
Q	4.961	4.954	4.948	4.934	4.918
DL		1.608	1.608	1.352	1.364
DR	2.740	2.736	2.732	2.725	2.716
SNR		0.007	0.007	0.013	0.016
SR		0.715	0.715	0.601	0.568
DA	3.279	3.279	3.279	3.279	3.279
X		0.002	0.002	0.003	0.003

This analysis examines the sensor performance across three different frequency bands, as presented in Tables 3 and 4, and 5. Across all tables, the refractive indices range from 1.33 to 1.36 RIU, representing different gaseous environments. The sensor’s sensitivity shows marked improvement as the frequency increases. In the lowest frequency band (Table 3), sensitivity ranges from 83 to 222 GHzRIU^− 1^. The middle frequency band (Table 4) maintains a consistent sensitivity of 222 GHzRIU^− 1^ for most of the range, dropping slightly to 167 GHzRIU^− 1^ at the highest refractive index. The highest frequency band (Table 5) demonstrates the best sensitivity, ranging from 417 to 444 GHzRIU^− 1^. This trend indicates that the sensor is more sensitive to refractive index changes at higher frequencies. The FWHM remains constant within each frequency band but varies across bands. It’s 0.276 THz in the lowest band, decreases to 0.132 THz in the middle band, and increases to 0.305 THz in the highest band. The narrower FWHM in the middle frequency band suggests better spectral resolution in this range. The FOM improves significantly with increasing frequency. In the lowest band, it ranges from 0.302 to 0.805 RIU^− 1^. The middle band shows a substantial improvement, with FOM values between 1.263 and 1.684 RIU^− 1^. The highest band maintains this improvement, with FOM values from 1.366 to 1.457 RIU^− 1^. This trend indicates better overall sensor performance at higher frequencies. The Q-factor shows a considerable improvement with increasing frequency. It ranges from 0.543 to 0.558 in the lowest band, increases significantly to 5.924–5.970 in the middle band, and slightly decreases to 4.918–4.961 in the highest band. The middle frequency band appears to offer the best balance of high Q-factor and other performance metrics. The DL varies across frequency bands, with the lowest band showing the best (lowest) values ranging from 0.012 to 0.033. The middle and highest bands have significantly higher DL values, ranging from 1.129 to 1.505 and 1.352 to 1.608, respectively. This suggests that while higher frequencies offer better sensitivity, they may sacrifice some ability to detect minute changes in refractive index. The DR improves with increasing frequency. It ranges from 0.286 to 0.293 in the lowest band, increases to 2.152–2.169 in the middle band, and further improves to 2.716–2.740 in the highest band. This indicates a wider operational range at higher frequencies. These parameters show varying trends across frequency bands. The SNR and SR generally improve with increasing frequency, indicating better signal quality and resolution. The uncertainty remains low across all bands but increases slightly in the highest frequency range. The DA varies across frequency bands, with the middle band showing the highest value of 7.576, compared to 3.623 in the lowest band and 3.279 in the highest band. This suggests that the middle frequency range offers the best detection accuracy. Table 6 show the comparative analysis.

**Table 6:** Comparison of the proposed sensor with existing studies in the literature.

	S(GHz/RIU)	DA	Q	Materials	Application
^46^	344∘RIU^− 1^	3.34	166.99	AgCl, Bp	Formalin detection
^29^	20,400 GHzRIU^− 1^	19.607	9.372	Graphene and Au	Alcohol detection
^47^	155.33° RIU^− 1^	0.62973	21	ZnO-Ag-PtSe2-Graphene	Formalin detection
^48^	508 GHzRIU^− 1^	22.22	5.089	Graphene	Proteins
^49^	33.98^− 1^° RIU^− 1^	0.2987	2.78019	Graphene Coating	pseudomonas-like bacteria detection
^50^	200GHzRIU^− 1^	18.5	9.09	Graphene and Au	THz sensing
^51^	98.00° RIU^− 1^	0.8889	88.8889	Ag, Graphene	Detection of Formalin
^52^	1773 GHzRIU^− 1^	30.303	21.727	Ag, Au and graphene	Fuel adulteration
^53^	250.2° RIU^− 1^	-	-	InP and Bp	Detection of Formalin
^54^	3500nmRIU^− 1^	5	18.292	Au, Ag and graphene	Hemoglobin
^55^	0.186 Abs (mg/L)^−1^	-	-	α-Fe_2_O_3_/ITO bioelectrode	Detection of Formalin.
^56^	400GHzRIU^− 1^	2.041	1.554	Mxene and graphene	Gas sensing
**Proposed sensor**	444GHzRIU^− 1^	7.576	5.970	Au, Bp, BaTiO_3_ Graphene	Detection of Formalin

Table 6 presents a comprehensive comparison of our proposed sensor’s performance against similar sensors reported in existing studies. Key metrics, including sensitivity (S), detection accuracy (DA), and quality factor (Q), highlight the improvements and unique characteristics of the proposed sensor. Unlike other sensors, which often use limited material combinations, the proposed sensor integrates advanced nanomaterials—black phosphorus (Bp), barium titanate (BaTiO3), gold (Au), and graphene.

### Encoding

**Fig. 10:**
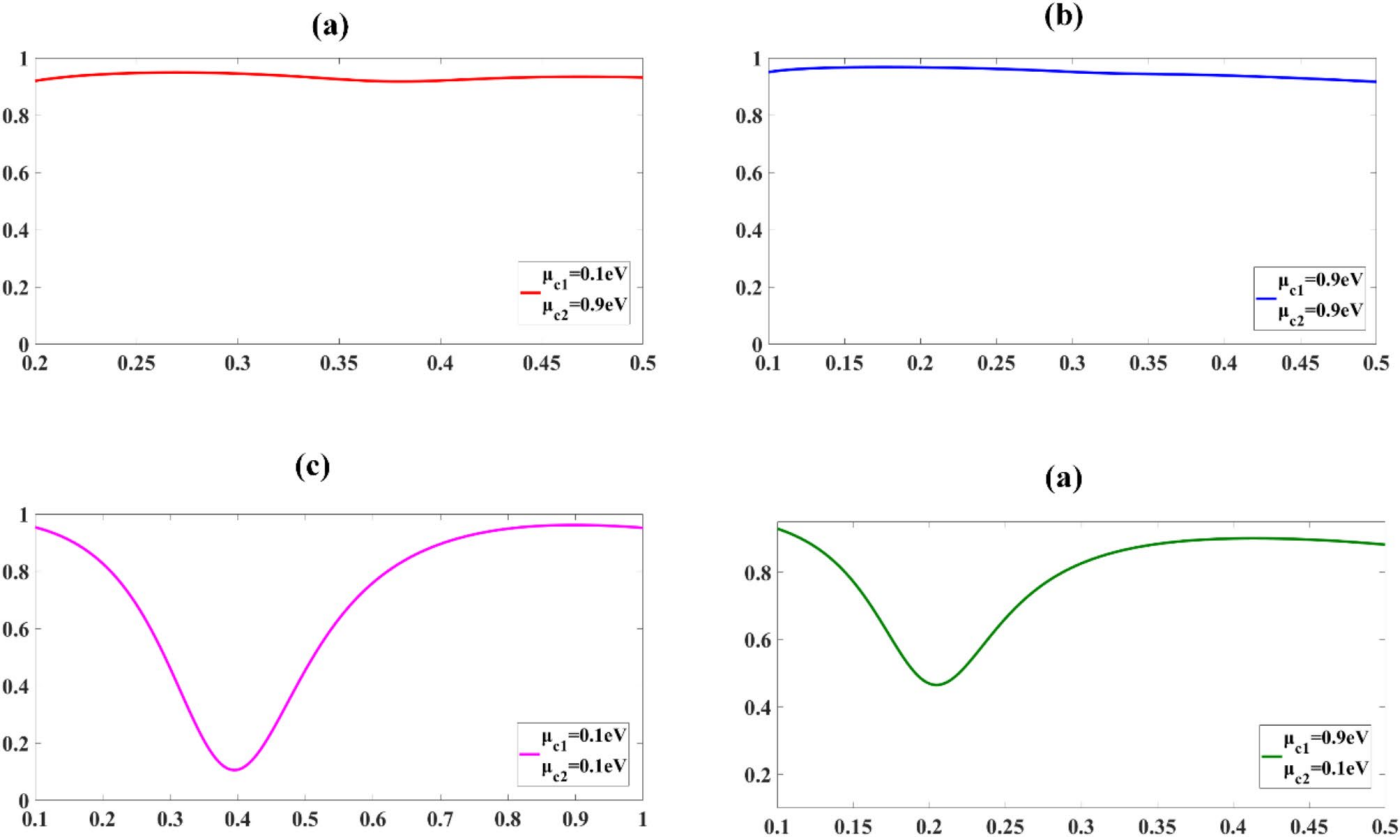
Transmittance response of the 2-bit sensor for various GCP combinations, illustrating the 2-bit encoding states.

Figure 10 presents analysis of the encoding capabilities of the proposed sensor, demonstrating its response to varying input parameters. The sensor’s performance is characterized by its transmittance properties under different combinations of chemical potentials (µ_c1_ and µ_c2_). In configurations where µ_c1_ = 0.1 eV and µ_c2_ = 0.9 eV (Fig. 10a), as well as µ_c1_ = 0.9 eV and µ_c2_ = 0.9 eV (Fig. 10b), the sensor exhibits exceptional optical transparency. The transmittance in these cases approaches unity (T ≈ 1) across the entire measured spectral range, indicating minimal light absorption or scattering within the device. This near-perfect transmittance suggests a state of high conductivity in the sensor design. Conversely, when both inputs are set to lower chemical potentials, specifically µ_c1_ = 0.1 eV and µ_c2_ = 0.1 eV (Fig. 10c), or in the asymmetric case of µ_c1_ = 0.9 eV and µ_c2_ = 0.1 eV (Fig. 10d), the sensor demonstrates a marked decrease in transmittance.

In these scenarios, the transmittance drops to approximately 50% (0.5 ≤ T < 1) of the incident light intensity. This reduction in transmittance is attributable to increased light-matter interactions, possibly through mechanisms such as plasmon excitation or inter band transitions. The observed binary-like behaviour in transmittance can be exploited for 2-bit encoding purposes. By assigning a logical ‘0’ to the high transmittance state (T ≈ 1) and a logical ‘1’ to the low transmittance state (T ≈ 0.5), the sensor effectively functions as a quaternary logic element. This encoding scheme allows for the representation of four distinct states: 00, 01, 10, and 11, corresponding to the four input configurations presented in Fig. 10.

## Machine learning optimization using locally weighted linear regression (LWLR)

LWLR is a technique that is applied to estimate the locally linear model for data which have a non-linear relation. While traditional model, known as linear weighted regression predicts the entire data set with a single global model, LWLR aims at decomposing this same data and applying a linear model to each point of interest respectively^57^. This makes the model slightly more accurate since it can adjust to local specificities and patterns that a unique model cannot pick.

Starting with every data point, importance weights are calculated in the proportion with the distance to the required prediction point. Usually, points nearer to the target point are factored with higher weights as the information is more localized to the local model^58^. One of the well-known approaches that can be used for these weights’ assignment is a kernel function for which weights decrease with the distance. Using these weights, a weighted linear regression model is then obtained for the data in the vicinity of the target point and characterizes the data by its local structure rather than the tendencies of the global scale^59^. The fitted model is then used to estimate the exact value at the specific target point while Considering the Effects of Nearby Data Points. First of all, the transformative ability of LWLR is one of the strengths as it can incorporate complicated, non-linear relationship that a global linear model may overlook and it is helpful when the relationship is non stationary throughout the data region. However, LWLR can be rather time consuming because for making any prediction point a new model needs to be estimated. However, the specification of the bandwidth parameter of the kernel function defining the size of the local neighbourhood has a critical influence on the quality of the model and its selection is also problematic^60^.

The analysis will utilize non-linear regression, as outlined in Eqs. 33,34 etc., to fit a curved line for maximum precision. Loss function notation, J(θ), applies within the framework of linear regression.

28 $$\:\text{J}\left({\Theta\:}\right)={{\sum\:}_{i=1}^{m}\left({y}^{i}-{{\Theta\:}}^{T}{x}^{\left(i\right)}\right)}^{2}$$

The revised loss function is

29 $$\:\text{J}\left({\Theta\:}\right)={{\sum\:}_{i=1}^{m}{w}^{\left(i\right)}\left({y}^{i}-{{\Theta\:}}^{T}{x}^{\left(i\right)}\right)}^{2}$$

Where $\:{w}^{\left(i\right)}\:$ is defined by;

30 $$\:{w}^{\left(i\right)}={e}^{-\frac{{\left({x}^{\left(i\right)}-x\right)}^{T}\left({x}^{\left(i\right)}-x\right)}{2}}$$

The objective in this scenario is to produce a prediction. Each training cycle completion is denoted by x^i^, representing the relevant data. The output of the function is limited to the range of 0 to 1. When accounting for errors, the focus is on x^i^ values in close proximity to the specified x. The methodology employs a weighting mechanism, w^i^, which integrates a key hyperparameter symbolized by tau (τ). This parameter is essential for the computations. The revised weight is calculated using the following Eq. 

31 $$\:{w}^{\left(i\right)}={e}^{-\frac{{\left({x}^{\left(i\right)}-x\right)}^{T}\left({x}^{\left(i\right)}-x\right)}{{2\text{t}}^{2}}}$$

Given the availability of a closed-form solution, the parameter θ can be computed as:

32 $$\:\Theta=\text{X}\text{T}\:\left(\text{W}\text{X}\right)^{-1}\left(\text{X}^{\text{T}}\text{W}\text{Y}\right)$$

The coefficient of determination is given by;

33 $$\:{R}^{2}=\frac{{\sum\:}_{i=1}^{N}\left.{\left(Predicted\:Target\:Value\right.}_{i}-{Actual\:Target\:Value}_{i}\right)}{{\sum\:}_{i=1}^{N}{\left({Actual\:Target}_{i}-Average\:Target\:Value\right)}^{2}}$$

Detailed discussion of these equations are given by^61,62^.

**Fig. 11:**
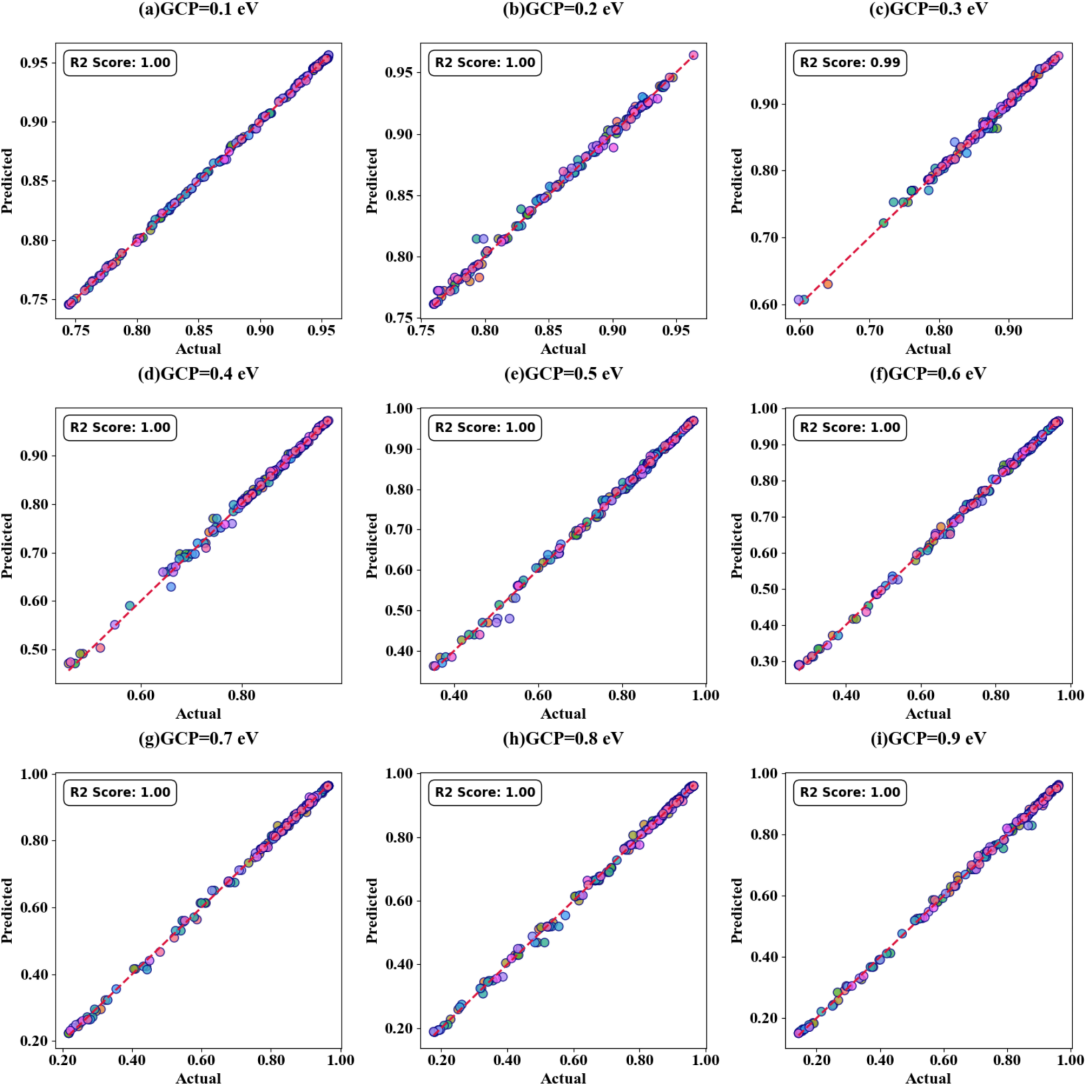
Scatter plots (SPs) illustrating the comparison between actual and predicted absorption values, demonstrating the high accuracy of the LOWESS regressor for GCP variation.

**Fig. 12:**
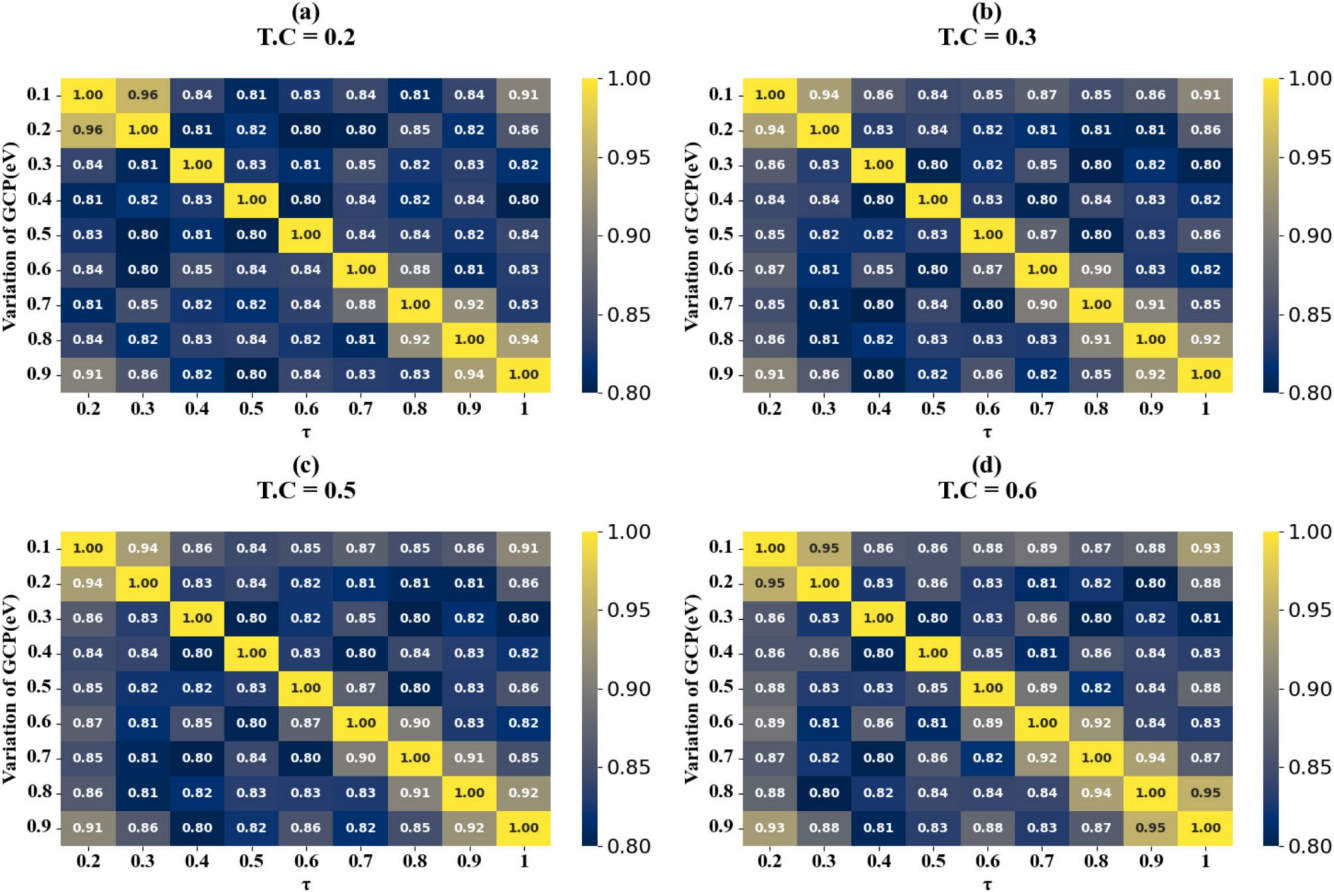
Heat map plots (HPs) illustrating the comparison between actual and predicted absorption values, demonstrating the high accuracy of the LOWESS regressor for GCP variation across different test cases.

**Fig. 13:**
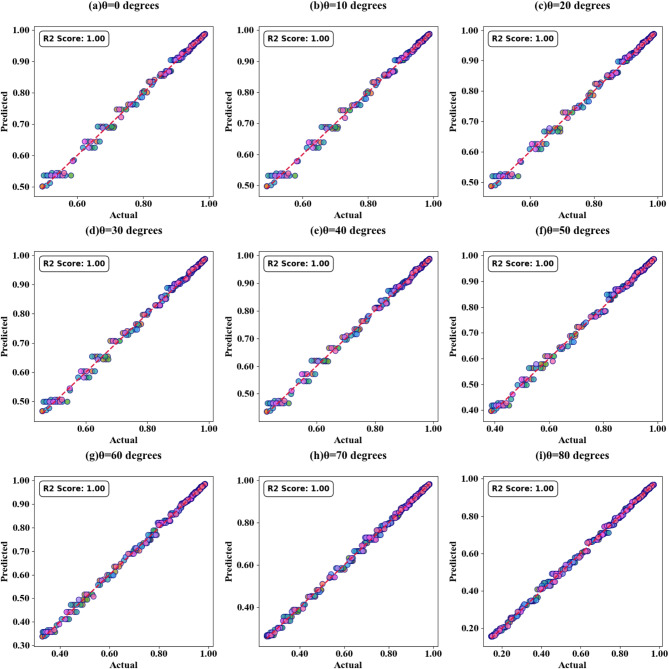
SPs illustrating the comparison between actual and predicted absorption values, demonstrating the high accuracy of the LOWESS regressor for θ(^o^) variation.

**Fig. 14:**
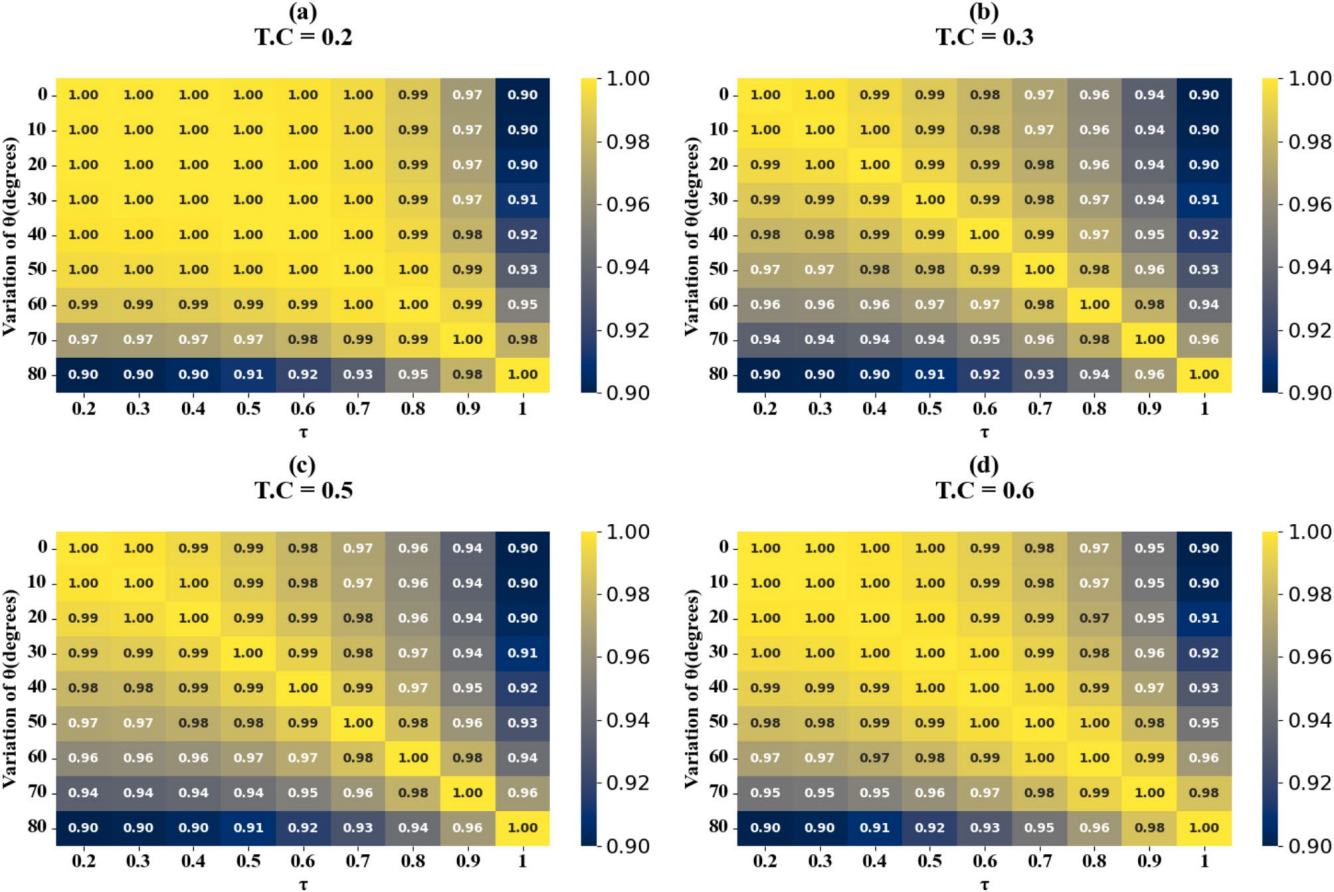
HPs illustrating the comparison between actual and predicted absorption values, demonstrating the high accuracy of the LOWESS regressor for θ(^o^) variation at the different test cases.

**Fig. 15:**
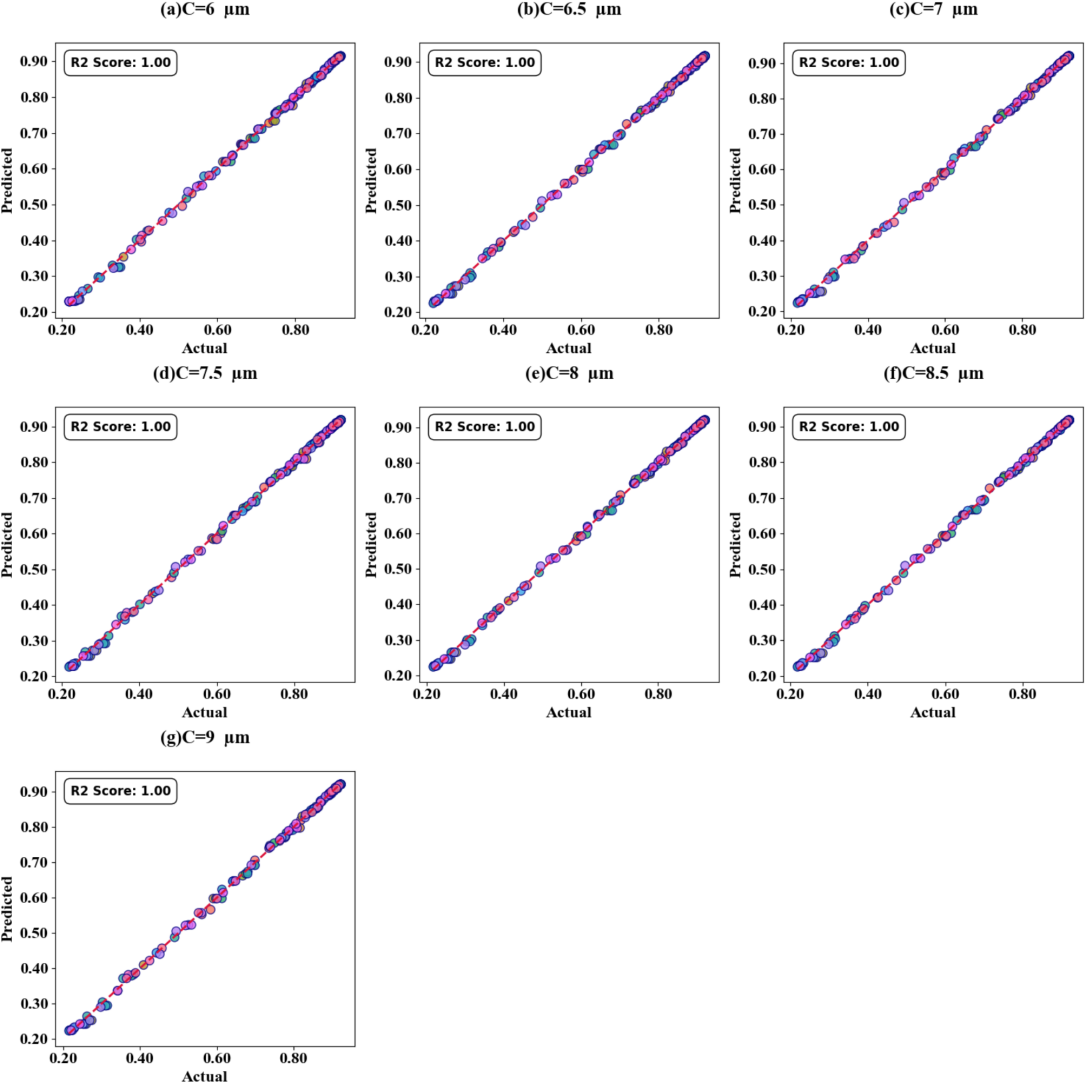
SPs illustrating the comparison between actual and predicted absorption values, demonstrating the high accuracy of the LOWESS regressor for Circular resonator variation.

**Fig. 16:**
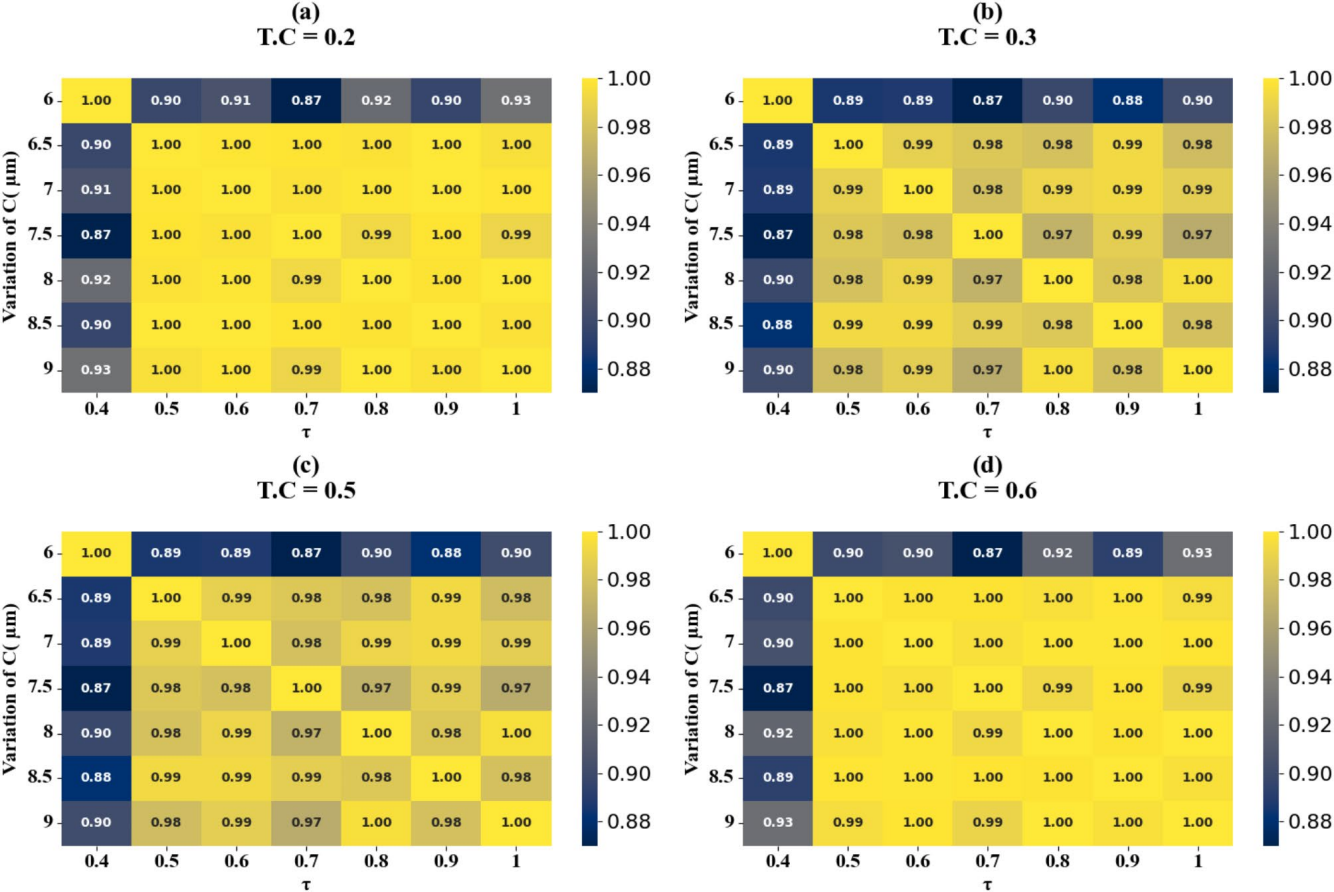
HPs illustrating the comparison between actual and predicted absorption values, demonstrating the high accuracy of the LOWESS regressor for Circular resonator variation at different test cases.

**Fig. 17:**
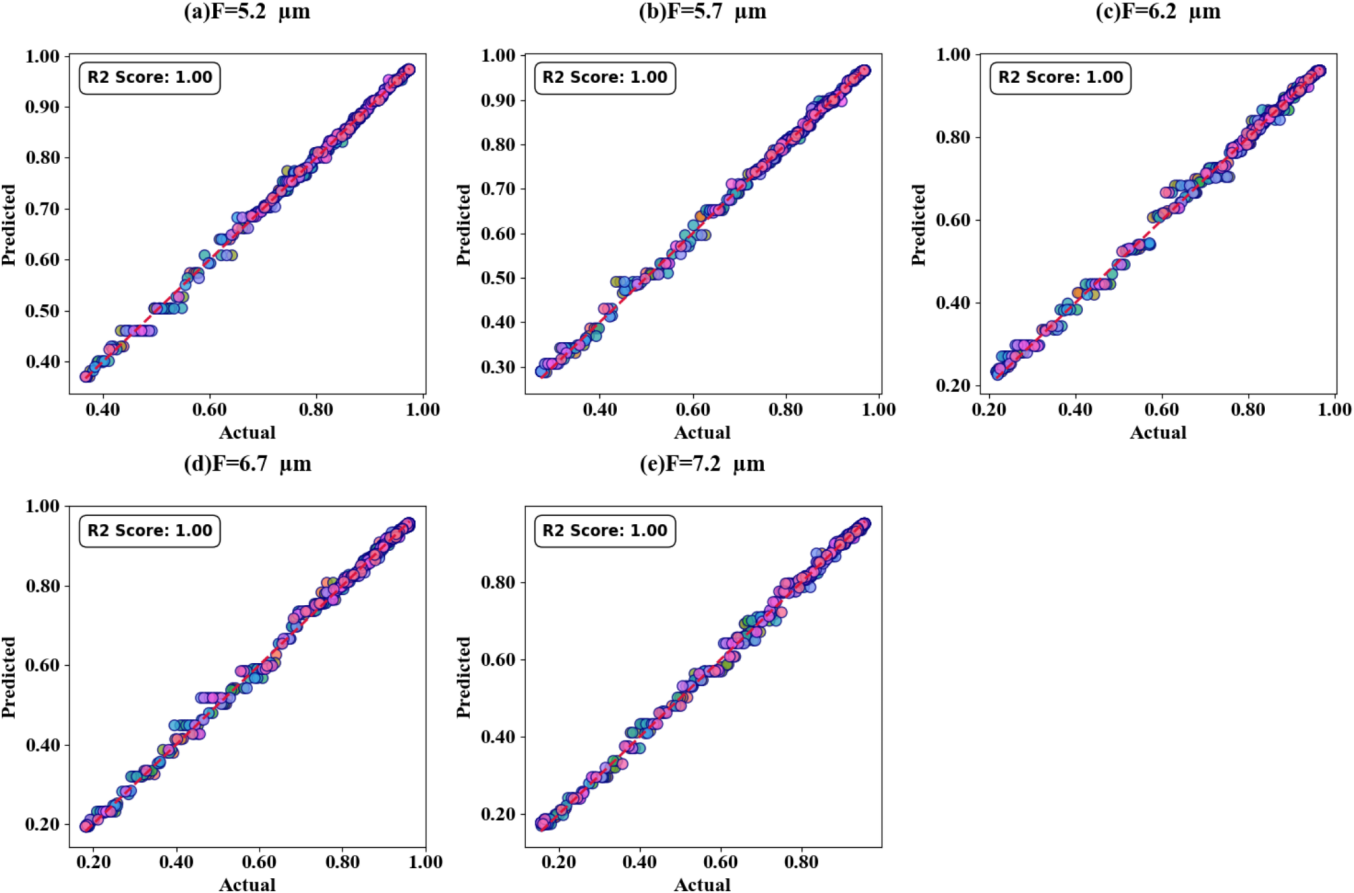
SPs illustrating the comparison between actual and predicted absorption values, demonstrating the high accuracy of the LOWESS regressor for the two Circular ring resonator variation.

**Fig. 18:**
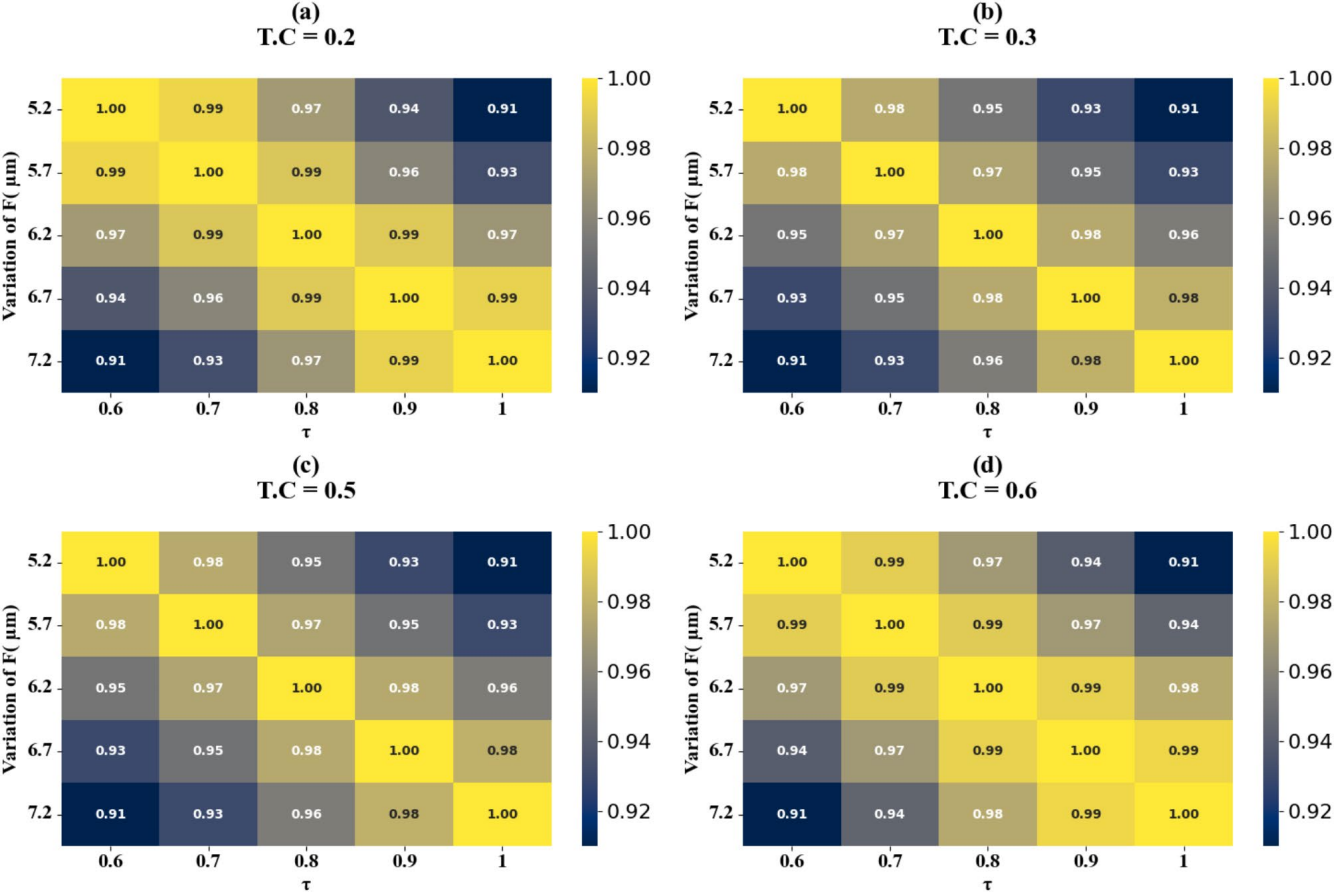
HPs illustrating the comparison between actual and predicted absorption values, demonstrating the high accuracy of the LOWESS regressor for two Circular ring resonator variation at different test cases.

**Fig. 19:**
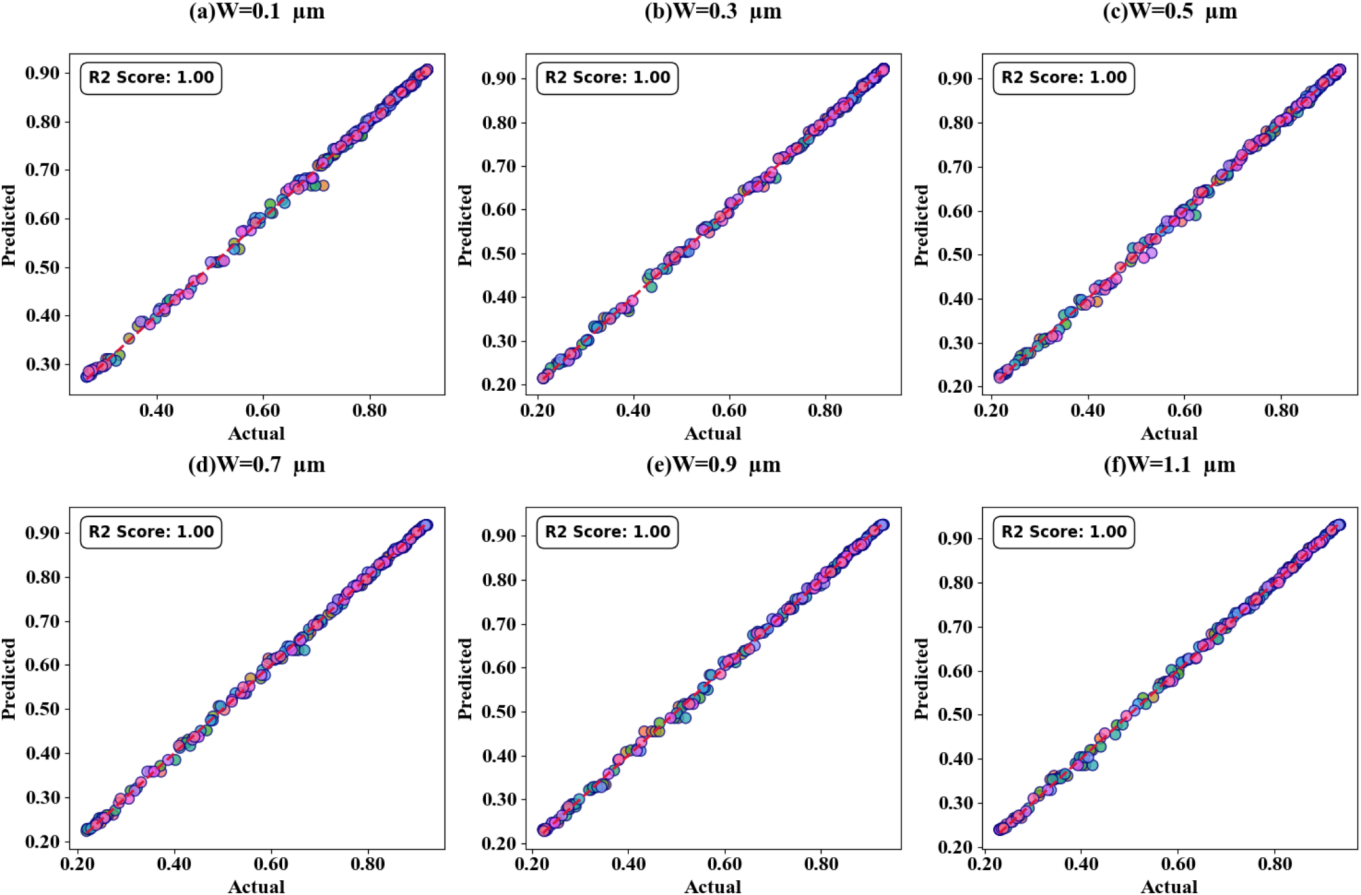
SPs illustrating the comparison between actual and predicted absorption values, demonstrating the high accuracy of the LOWESS regressor for the variation of the width of H shaped resonator .

**Fig. 20:**
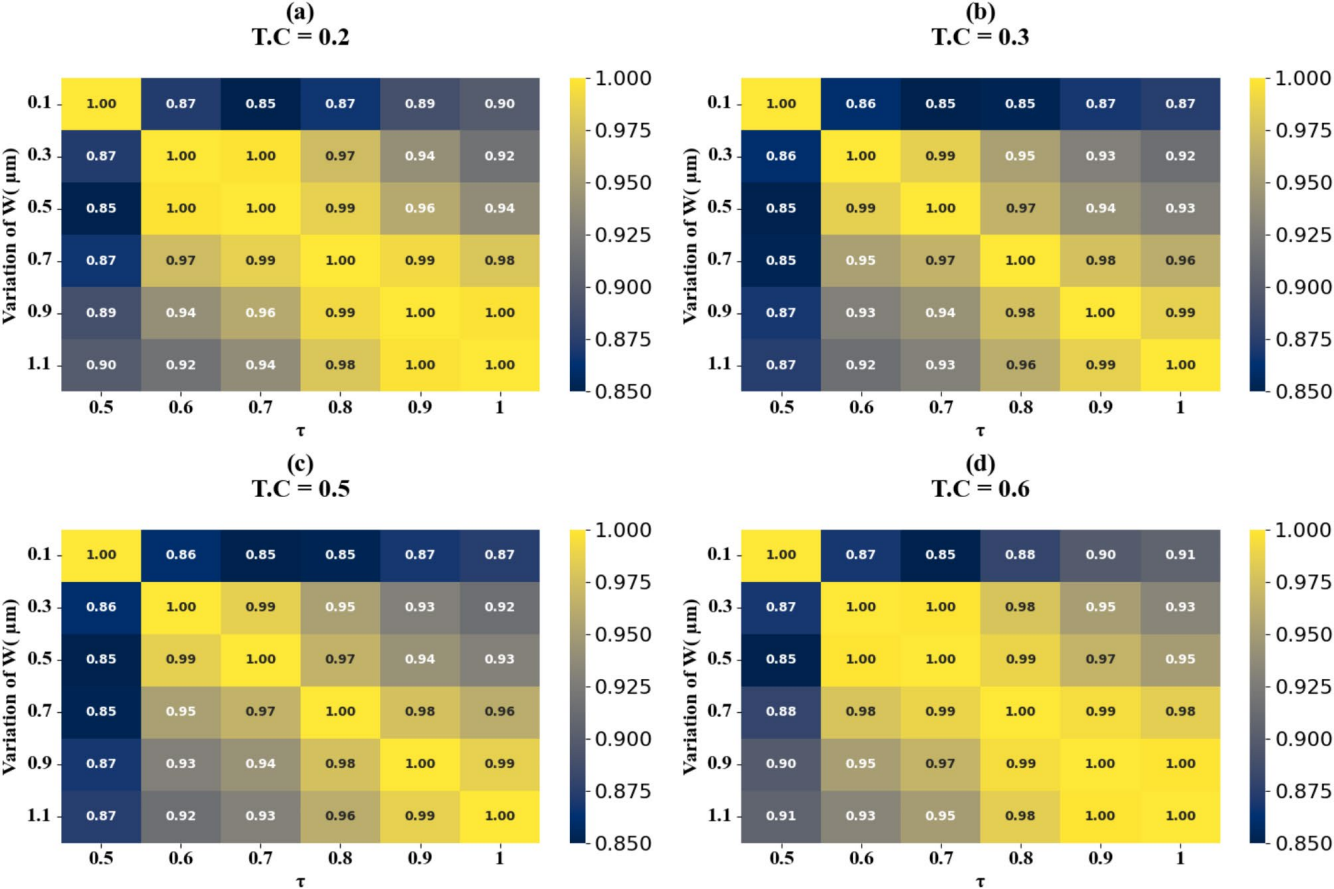
HPs illustrating the comparison between actual and predicted absorption values, demonstrating the high accuracy of the LOWESS regressor for the variation of the width of H shaped resonator at different test cases.

**Fig. 21:**
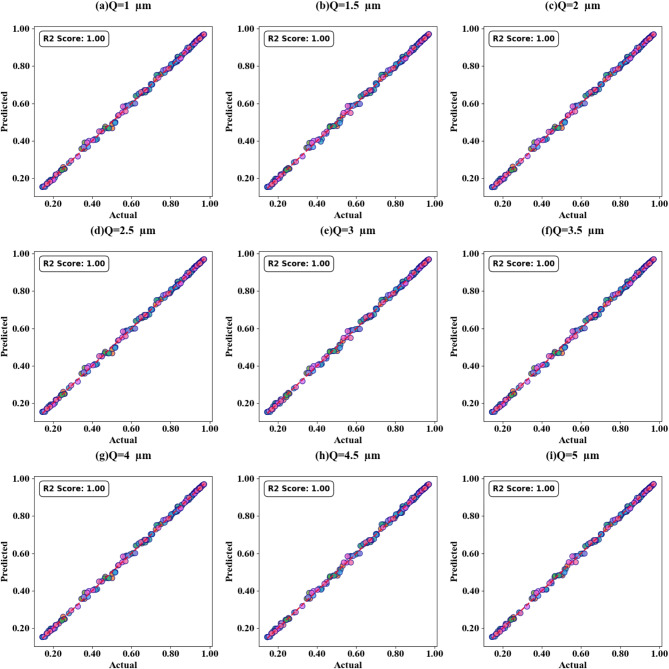
SPs illustrating the comparison between actual and predicted absorption values demonstrating the high accuracy of the LOWESS regressor for the variation of the Length of H shaped resonator.

**Fig. 22:**
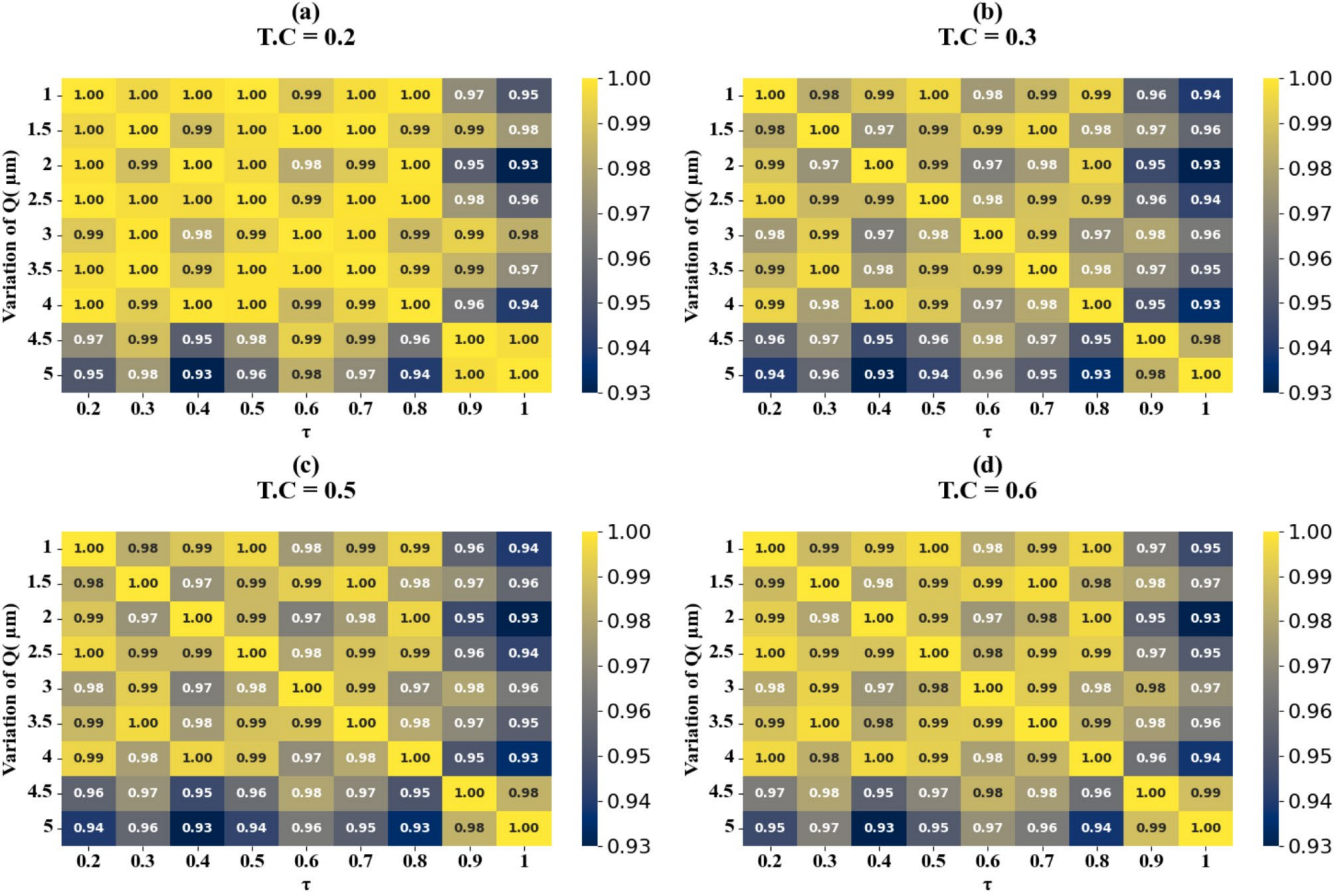
HPs illustrating the comparison between actual and predicted absorption values demonstrating the high accuracy of the LOWESS regressor for the variation of the length of H shaped resonator at different test cases.

**Fig. 23:**
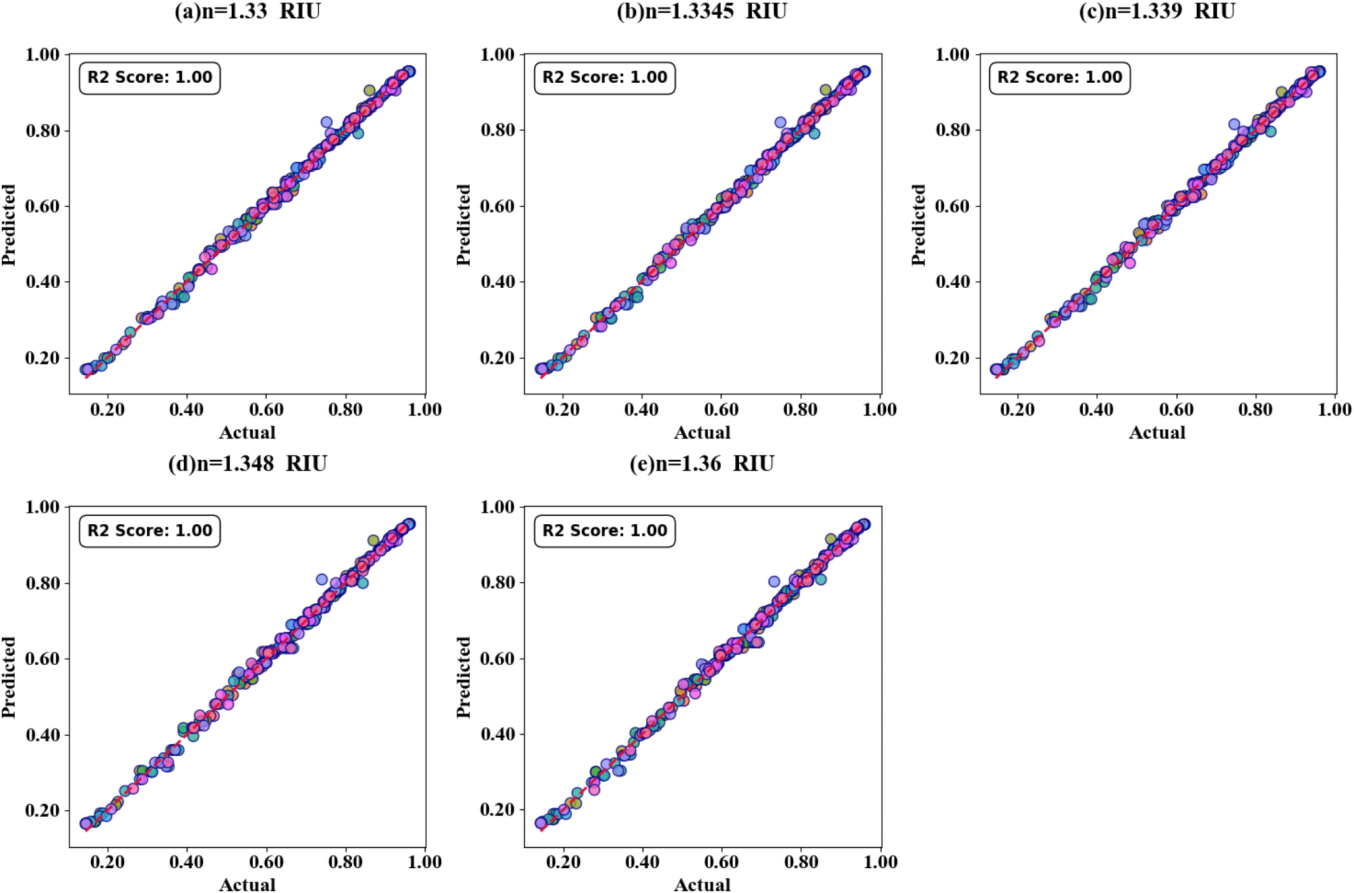
SPs illustrating the comparison between actual and predicted absorption values demonstrate the high accuracy of the LOWESS regressor for the variation of n(TC = 0.15).

**Fig. 24:**
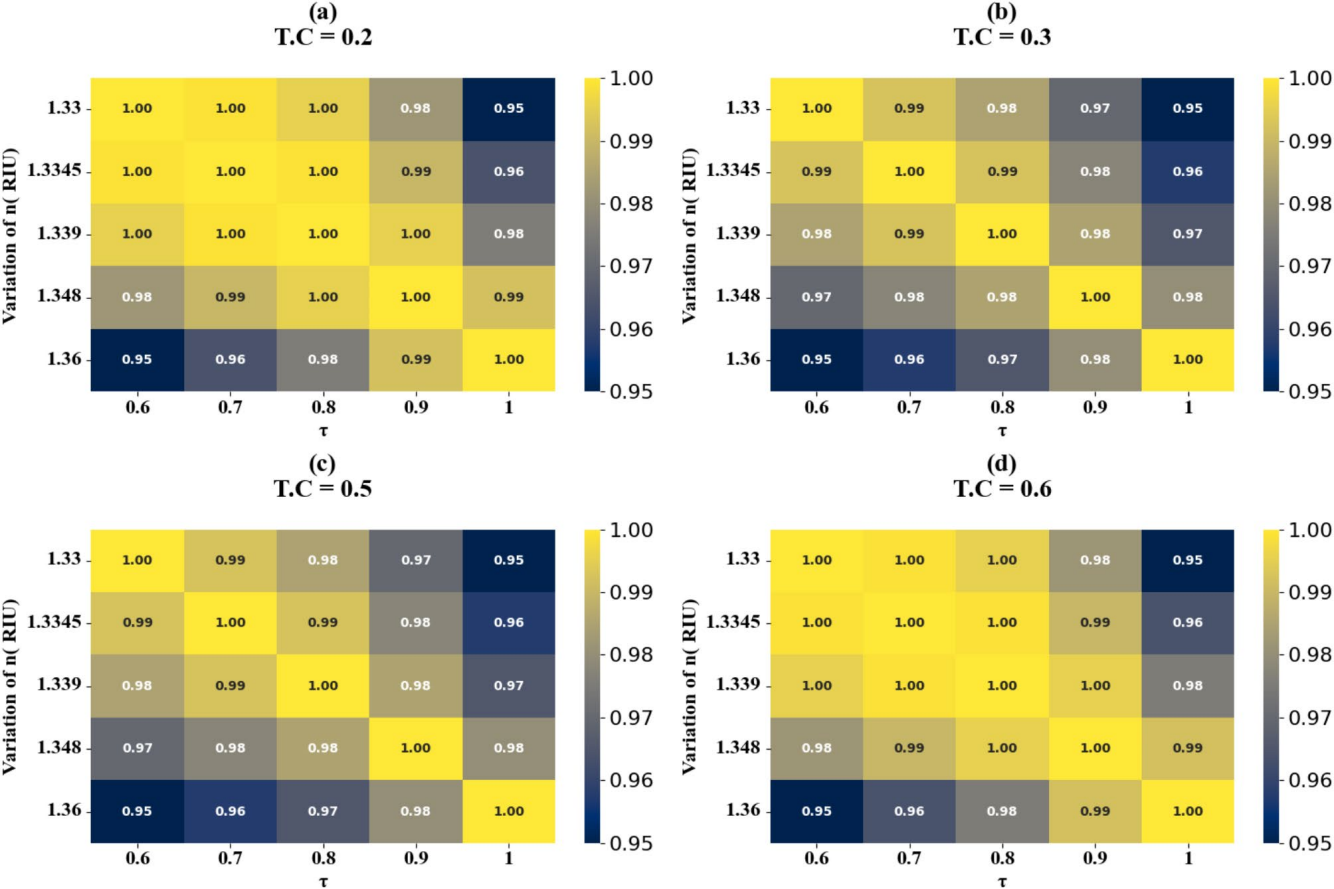
HPs illustrating the comparison between actual and predicted absorption values demonstrate the high accuracy of the LOWESS regressor for the variation of the n at different test cases.

Figure 11 (a-i) displays scatter plots depicting the correlation between GCP variation and prediction accuracy. These plots demonstrate the performance of the LOWESS regressor model in predicting absorption with high precision for GCP values spanning from 0.1 eV to 0.9 eV across multiple test cases (TC = 0.1, 0.15, 0.2, 0.35, 0.38, 0.4, 0.45, 0.49, and 0.5). The scatter plots consistently show that the model attains a maximum coefficient of determination (R²) score of 1 for all examined scenarios, thereby validating the model’s robust predictive capability. In the subsequent phase of the study, we investigated the impact of increasing τ (tau) values on the accuracy of absorption predictions. The results of this analysis are showcased in Fig. 12 (a to d), these findings clearly indicate that prediction accuracy is influenced by changes in τ values. Notably, we observed that certain τ values lead to enhanced prediction accuracy, while others result in decreased accuracy. Based on these observations, we can conclude that for optimal predictions, it is advisable to implement the LOWESS regressor model using τ values that correspond to higher prediction accuracy. This approach allows for fine-tuning of the model to achieve the best possible results across different GCP values, potentially improving the overall reliability and precision of absorption predictions in various scenarios.

Scatter plots illustrating the relationship between predicted absorption values and actual absorption values obtained using the LOWESS regressor are presented in Fig. 13 (a-i). These plots correspond to sensor incidence angles ranging from 0° to 80° and test cases (TC) of 0.2, 0.25, 0.27, 0.3, 0.31, 0.36, 0.38, 0.42, and 0.44. An optimal coefficient of determination (R²) of 1 is achieved across all cases examined. The effect of increasing τ values is visualized through heat map plots in Fig. 14a and d for test cases 0.2, 0.3, 0.5, and 0.6. Across all test cases, the R² score ranges from 0.91 to 1, indicating a high degree of correlation between predicted and actual absorption values. Figure 15 (a-g) present scatter plots showing the relationship between predicted and actual absorption values using the LOWESS regressor, for circular resonators varying from 6 μm to 9 μm and test cases of 0.32, 0.34, 0.36, 0.43, 0.45, and 0.44, achieving an R² of 1 in all cases. Figure 16a and d display heat maps illustrating the effect of increasing τ values for test cases 0.2, 0.3, 0.5, and 0.6, with R² scores ranging from 0.93 to 1.

Scatter plots in Fig. 17 (a-e) illustrate the correlation between predicted and actual absorption values for circular ring resonators. These plots use the LOWESS regressor and cover resonator sizes from 5.2 μm to 7.2 μm, with test cases of 0.42, 0.44, 0.46, 0.48, and 0.5. In all instances, a perfect R² score of 1 is achieved. Figure 18(a-d) show heat maps that demonstrate how increasing τ values affect test cases 0.2, 0.3, 0.5, and 0.6. The R² scores for these cases range from 0.85 to 1.

The scatter plots in Fig. 19 (a-f) show the correlation between predicted and actual absorption values for H-shaped resonators of varying widths, using the LOWESS regressor. These resonator dimensions range from 0.1 μm to 1.1 μm, with test cases at 0.51, 0.53, 0.57, 0.58, 0.59, and 0.6. Each case achieves an ideal R² score of 1. Figure 20a to d present heat maps illustrating the effect of increasing τ values on test cases 0.2, 0.3, 0.5, and 0.6, where the R² scores range between 0.95 and 1.

The correlation between predicted and actual absorption values for H-shaped resonators of varying lengths is depicted in scatter plots in Fig. 21 (a-i). These plots employ the LOWESS regressor and encompass resonator dimensions from 1 μm to 5 μm. Test cases include 0.21, 0.23, 0.26, 0.32, 0.34, 0.38, 0.41, 0.44, and 0.48. Each case achieves an ideal R² score of 1. Heat maps in Fig. 22a and d illustrate the impact of increasing τ values on test cases 0.2, 0.3, 0.5, and 0.6. The R² scores for these instances range between 0.93 and 1. Comparable results are observed for the variation of refractive indices, as shown in Figs. 23a to e and 24a to d. These findings demonstrate that employing the LOWESS regressor can reduce simulation time and resources by at least 78%.

## Conclusion

In summary, this study has introduced a biosensor proposed to detect formalin in water. The proposed sensor, operating in the terahertz regime, integrates advanced nanomaterials to enhance its sensitivity and performance. The analysis demonstrates the sensor’s capability to detect formalin across three distinct frequency bands, with the highest sensitivity of 444 GHzRIU^− 1^. The sensor exhibited excellent performance metrics, including Q-factor reaching 5.970, and a detection accuracy of 7. 576. The electrical field strength examination demonstrates the sensor’s absorption and transmission attributes exemplifying optimal functioning at 0.8 THz. Moreover, the sensor also showcases promise for 2-bit encoding uses, highlighting its adaptability beyond formalin identification. Moreover, this research utilized a machine learning technique, specifically Locally Weighted Linear Regression (LWLR), to enhance the sensor’s efficacy and significantly reduce computational time. This method proved highly effective, cutting simulation duration by nearly four-fifths while maintaining exceptional accuracy in predicting absorption across diverse sensor parameters. When compared to previously published research, the proposed sensor design exhibited markedly improved sensitivity and overall performance metrics. These advancements position the proposed device as a promising candidate for detecting formalin in both environmental and healthcare contexts. Moving forward, research efforts could concentrate on improving the sensor’s ability to distinguish between similar substances, as well as evaluating its potential to identify other hazardous compounds. Additionally, assessing its effectiveness in non-laboratory settings would provide valuable insights into its real-world applicability. Also, integrating this sensing technology into compact, mobile devices would enable immediate, on-location formalin detection across various applications.

## Data Availability

The data used to support the findings of this study are included in the article.
